# FasL^+^PD‐L2^+^ Identifies a Novel Immunosuppressive Neutrophil Population in Human Gastric Cancer That Promotes Disease Progression

**DOI:** 10.1002/advs.202103543

**Published:** 2021-12-26

**Authors:** Zhi‐Guo Shan, Yong‐Liang Zhao, Jin‐Yu Zhang, Zong‐Bao Yan, Ting‐Ting Wang, Fang‐Yuan Mao, Yong‐Sheng Teng, Liu‐Sheng Peng, Wan‐Yan Chen, Pan Wang, Ping Cheng, Wen‐Qing Tian, Jun Chen, Weisan Chen, Yuan Zhuang

**Affiliations:** ^1^ Department of General Surgery and Center of Minimal Invasive Gastrointestinal Surgery Southwest Hospital Third Military Medical University Chongqing 400038 China; ^2^ National Engineering Research Center of Immunological Products Department of Microbiology and Biochemical Pharmacy College of Pharmacy and Laboratory Medicine Third Military Medical University Chongqing 400038 China; ^3^ Chongqing Key Research Laboratory for Drug Metabolism Department of Pharmacology Chongqing Medical University Chongqing 400016 China; ^4^ Department of Endocrinology the First Affiliated Hospital of Chongqing Medical University Chongqing 400016 China; ^5^ La Trobe Institute of Molecular Science La Trobe University Bundoora Victoria 3085 Australia; ^6^ Department of Gastroenterology the Affiliated Hospital of Southwest Medical University Luzhou Sichuan 646000 China; ^7^ Jiangsu Key Laboratory of Medical Science and Laboratory Medicine School of Medicine Jiangsu University Jiangsu 21013 China; ^8^ Department of Gastroenterology Southwest Hospital Third Military Medical University Chongqing 400038 China; ^9^ Department of Gastroenterology the Affiliated Hospital of Zunyi Medical University Zunyi Guizhou 563003 China

**Keywords:** FasL, gastric cancer, neutrophils, PD‐L2

## Abstract

Neutrophils constitute abundant cellular components in human gastric cancer (GC) tissues, but their protumorigenic subset in pathogenesis of GC progression is unclear. Here, it is found that patients with GC show significantly higher neutrophil infiltration in tumors that is regulated by CXCL12‐CXCR4 chemotaxis. These tumor‐infiltrating neutrophils express high level immunosuppressive molecules FasL and PD‐L2, and this FasL^+^PD‐L2^+^ neutrophil subset with a unique phenotype constitutes at least 20% of all neutrophils in advanced GC and predicts poor patient survival. Tumor induces neutrophils to express FasL and PD‐L2 proteins with similar phenotype to those in GC tumors in both time‐dependent and dose‐dependent manners. Mechanistically, Th17 cell‐derived IL‐17A and tumor cell‐derived G‐CSF can significantly induce neutrophil FasL and PD‐L2 expression via activating ERK‐NF‐*κ*B and JAK‐STAT3 signaling pathway, respectively. Importantly, upon over‐expressing FasL and PD‐L2, neutrophils acquire immunosuppressive functions on tumor‐specific CD8^+^ T‐cells and promote the growth and progression of human GC tumors in vitro and in vivo, which can be reversed by blocking FasL and PD‐L2 on these neutrophils. Thus, the work identifies a novel protumorigenic FasL^+^PD‐L2^+^ neutrophil subset in GC and provides new insights for human cancer immunosuppression and anti‐cancer therapies targeting these pathogenic cells.

## Graphical abstract

CXCL12‐CXCR4 chemotaxis mediates the recruitment of neutrophils into gastric cancer (GC) environment. Th17 cell‐derived IL‐17A and tumor cell‐derived G‐CSF activate ERK‐NF‐*κ*B and JAK‐STAT3 signaling pathways to induce FasL and PD‐L2 expression on neutrophils. These immunosuppressive FasL^+^PD‐L2^+^ neutrophils exert protumorigenic effects by suppressing tumor‐specific CD8^+^ T‐cell immunity in FasL/PD‐L2 dependent manners. Targeting these pathological FasL^+^PD‐L2^+^ neutrophils will improve GC treating.

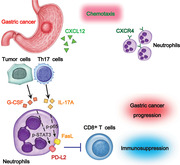

## 1 Introduction

Tumor progression is now recognized as the result of evolving cross‐talks among different cell types within the tumor environment, which creates an immunosuppressive network to promote immune evasion and tumor growth.^[^ ^1^ ^]^ Various immune cells have been reported to infiltrate in the tumor environment;^[^ ^2^ ^]^ among them, neutrophils often constitute the most abundant component of infiltrated leukocytes.^[^ ^3^ ^]^ Although less well characterized than tumor‐associated macrophages,^[^ ^4^ ^]^ tumor‐infiltrating neutrophils are emerging as an important player in the pathophysiology of tumor progression.^[^ ^5^ ^]^

Gastric cancer (GC), as one of most common malignancies, has been the second leading cause of cancer death worldwide.^[^ ^6^ ^]^ It has been reported that a close relation exists between increased tumor‐infiltrating neutrophils and poor GC patient prognosis,^[^ ^7^ ^]^ suggesting that neutrophils could be potential therapeutic targets for GC. It has also been reported that elevated neutrophil/lymphocyte ratio in peripheral blood of GC patients predicts poor patient survival,^[^ ^8^ ^]^ suggesting that neutrophils might have potential promoting roles on GC by mediating T cell dysfunction. Therefore, characterizing novel neutrophil subsets, and elucidating their immune regulatory and suppressive mechanisms in GC tumors are essential for understanding their potential protumorigenic roles in tumor immunopathogenesis.

Herein, we show that a novel FasL^+^PD‐L2^+^ neutrophil subset that constitutes more than 20% of all neutrophils in advanced GC exhibits a unique phenotype differing from that of the conventional FasL^−^PD‐L2^−^ peripheral neutrophils and, that their infiltration in GC is associated with disease progression and is negatively correlated with patient survival following surgery. Moreover, we demonstrate mechanistically that T helper (Th) 17 cell‐derived interleukin (IL)‐17A and tumor cell‐derived granulocyte‐colony stimulating factor (G‐CSF) significantly induce neutrophil FasL and PD‐L2 expression via activating extracellular signal‐regulated kinase (ERK)‐nuclear factor *κ*B (NF‐*κ*B) and Janus kinase (JAK)‐signal transducer and activator of transcription 3 (STAT3) signaling pathway respectively. In turn, these FasL^+^PD‐L2^+^ neutrophils suppress tumor‐specific CD8^+^ T‐cell immunity and promote the growth and progression of human GC tumors.

## 2 Results

### 2.1 Increased Infiltration of Neutrophils in GC Is Correlated with Disease Stage and Poor Survival in Patients

**Figure 1:**
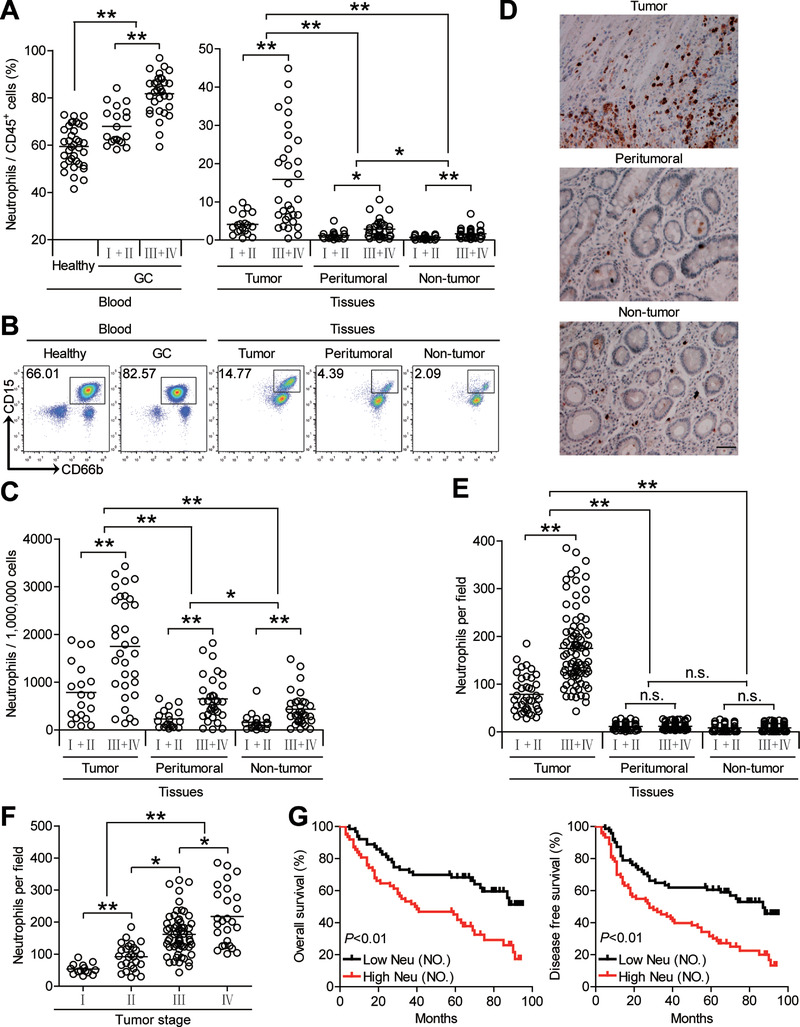
Increased infiltration of neutrophils in GC is correlated with disease stage and poor survival in patients. A,C) Neutrophil percentage in CD45^+^ cells or the total number of neutrophils per million total cells among TNM stages (I+II vs III+IV) in each tissue of patients with GC by gating on CD45^+^CD11b^+^CD66b^+^CD15^+^ cells or counting. Cumulative results from 51 GC patients and 36 healthy donors are shown. B) Dot plots of surface molecule staining for neutrophils gating on CD45^+^CD11b^+^ cells. D) Representative analysis of CD15^+^ (brown) neutrophil distributions in tissues of GC patients by immunohistochemical staining. Scale bars: 100 µm. E) Neutrophil number among TNM stages (I+II vs III+IV) in each tissue of patients with GC by immunohistochemical staining and counting. Cumulative results from 125 GC patients were shown. F) Neutrophil number among TNM stages was compared. G) Kaplan‐Meier plots for overall survival and disease‐free survival by median neutrophil number (132 per field). Data are mean ± SEM and analyzed by Student's *t*‐test, Mann‐Whitney U‐test, and one‐way ANOVA. *P < 0.05, **P < 0.01, n.s. *P* > 0.05 for groups connected by horizontal lines. Neu (NO), neutrophil number.

To identify neutrophil subsets in human GC, we first used flow cytometry to analyze the percentage of all neutrophils within the total CD45^+^ leukocytes by gating on CD45^+^CD11b^+^CD66b^+^CD15^+^ cells in different samples from 51 GC patients (Cohort 1). Peripheral blood samples from healthy donors were used as controls. Notably, patients with GC showed a higher neutrophil percentage in peripheral blood than healthy donors. Within the patient cohort, tumors contained a significantly higher neutrophil percentage than peritumoral and non‐tumor tissues (**Figure** **1**A,B). Moreover, as the cancer progressed, we found that the percentage of neutrophils significantly increased in each of the tested samples (Figure 1A). Similar observations were made when analyzing the total number of neutrophils per million total cells in each tissue (Figure 1C). Next, we used immunohistochemistry to analyze neutrophil infiltration in different tissue samples from 125 GC patients (Cohort 2). Immunohistochemical staining also showed that neutrophils accumulated in tumors (Figure 1D,E), and such accumulation was most noticeable from stage I onwards (Figure 1F). In keeping with these findings, increased neutrophil percentage and neutrophil number were correlated with increased tumor size, advanced tumor stage and advanced lymphatic invasion (Figures S1 and S2, Supporting Information).

Next, we evaluated the clinical relevance of intratumoral neutrophils in GC. Comparing patients (Cohort 2) with high (≥132 median level) versus low (<132) neutrophil number per field, the 95‐month overall survival and disease‐free survival rates were significantly lower for those within the higher neutrophil number group (Figure 1G). Importantly, the finding that intratumoral neutrophil number independently predicted survival was verified by multivariate analyses using a Cox proportional hazard model (Tables S1 and S2, Supporting Information). Taken together, these findings suggest that increased intratumoral neutrophil infiltration is associated with tumor progression and poor survival of GC patients.

### 2.2 FasL^+^PD‐L2^+^ Neutrophil Subset with a Unique Phenotype Is Increased in GC as Tumor Progresses and Predicts Poor Patient Survival

**Figure 2:**
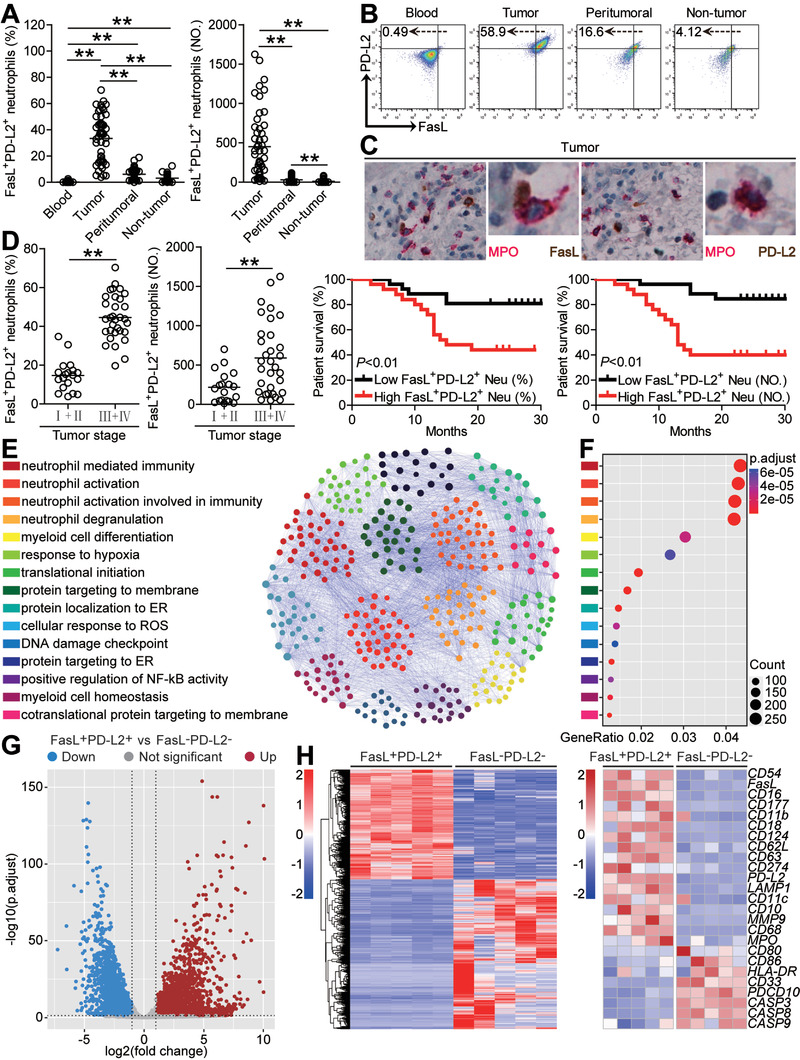
FasL^+^PD‐L2^+^ neutrophil subset with a unique phenotype is increased in GC as tumor progresses and predicts poor patient survival. A) Statistics analysis of FasL^+^PD‐L2^+^ neutrophil percentage in total neutrophils or FasL^+^PD‐L2^+^ neutrophil number per million total cells in each samples of patients with GC (*n* = 51). B) Dot plots of surface molecule staining for FasL^+^PD‐L2^+^ neutrophils gating on CD45^+^CD11b^+^CD66b^+^CD15^+^ cells. C) Representative image of MPO^+^FasL^+^ neutrophils and MPO^+^PD‐L2^+^ neutrophils in tumor tissues of GC patients by immunohistochemical staining. D) FasL^+^PD‐L2^+^ neutrophil percentage and FasL^+^PD‐L2^+^ neutrophil number among TNM stages was compared. Kaplan‐Meier plots for overall survival by median FasL^+^PD‐L2^+^ neutrophil percentage (36.8) and FasL^+^PD‐L2^+^ neutrophil number (306). E) PPI network analysis of significantly changed genes from top 15 gene ontology terms in tumor‐infiltrating FasL^+^PD‐L2^+^ neutrophils, as compared with peripheral FasL^−^PD‐L2^−^ neutrophils. F) Gene ontology analysis of significantly changed genes in tumor‐infiltrating FasL^+^PD‐L2^+^ neutrophils, as compared with peripheral FasL^−^PD‐L2^−^ neutrophils. G) Differentially expressed genes between tumor‐infiltrating FasL^+^PD‐L2^+^ neutrophils and peripheral FasL^−^PD‐L2^−^ neutrophils are shown. H) Heatmap revealing gene changes between tumor‐infiltrating FasL^+^PD‐L2^+^ neutrophils and peripheral FasL^−^PD‐L2^−^ neutrophils from five GC patients. Data are mean ± SEM and analyzed by Student's *t*‐test, Mann‐Whitney U‐test, and one‐way ANOVA. *P < 0.05, **P < 0.01 for groups connected by horizontal lines. MPO, myeloperoxidase; FasL^+^PD‐L2^+^ Neu (%), FasL^+^PD‐L2^+^ neutrophil percentage; FasL^+^PD‐L2^+^ Neu (NO), FasL^+^PD‐L2^+^ neutrophil number.

Immunosuppressive cells possessing immunoinhibitory signals and/or apoptotic signals have been known as potential promoters of tumor progression. The interactions of PD‐1‐PD‐L2^[^ ^9^, ^10^ ^]^ or Fas‐FasL^[^ ^11^, ^12^ ^]^ are the major mechanisms contributing to such immunosuppressive effects. To see whether similar phenotypes might be exhibited on the neutrophils in GC, we first examined the expressions of PD‐L2 and FasL, and found that intratumoral neutrophils expressed significantly higher immunosuppressive molecules FasL and PD‐L2 than those from peritumoral and non‐tumor tissues whereas peripheral neutrophils expressed little FasL nor PD‐L2 (Figure S3A–C, Supporting Information). Moreover, significant correlations were found between the levels of FasL and PD‐L2 expression on neutrophils in tumors analyzed (Figure S3D, Supporting Information), suggesting simultaneous co‐expressing of FasL and PD‐L2 on tumor‐infiltrating neutrophils. Importantly, to evaluate the potential subsets of infiltrating neutrophils in human GC, we found that GC patients showed a higher FasL^+^PD‐L2^+^ neutrophil subset in their tumor tissues than that in the blood, peritumoral and non‐tumor tissues (**Figure** **2**A,B). Moreover, as the cancer progressed, this FasL^+^PD‐L2^+^ neutrophil subset increased significantly (Figure 2D). Comparing patients (Cohort 1) with high (≥36.8 median level) versus low (<36.8) FasL^+^PD‐L2^+^ neutrophil percentage or with high (≥306 median level) versus low (<306) FasL^+^PD‐L2^+^ neutrophil number, the 30‐month overall survival rates were significantly lower for those within the higher FasL^+^PD‐L2^+^ neutrophil group (Figure 2D). In keeping with these findings, increased FasL^+^PD‐L2^+^ neutrophil percentage and FasL^+^PD‐L2^+^ neutrophil number correlated with increased tumor size and advanced tumor stage (Figure S4, Supporting Information). Importantly, the finding that intratumoral FasL^+^PD‐L2^+^ neutrophil number independently predicted survival was verified by multivariate analyses using a Cox proportional hazard model (Table S3, Supporting Information). RNA sequencing (RNA‐seq) analyses and gene ontology (GO) analysis indicated that FasL^+^PD‐L2^+^ neutrophils showed amplified the activation, degranulation, and immunity networks, compared to those in peripheral FasL^−^PD‐L2^−^ neutrophils (Figure 2E,F), but significantly decreased activities associated with lymphocyte activation and apoptosis (Figure 2G,H). Taken together, the above data indicate that FasL^+^PD‐L2^+^ neutrophils with a unique phenotype are increased in GC as tumor progresses and predict poor patient survival.

### 2.3 Human GC Environments Contribute to Neutrophil Infiltration and Induce FasL^+^PD‐L2^+^ Neutrophil Subset

**Figure 3:**
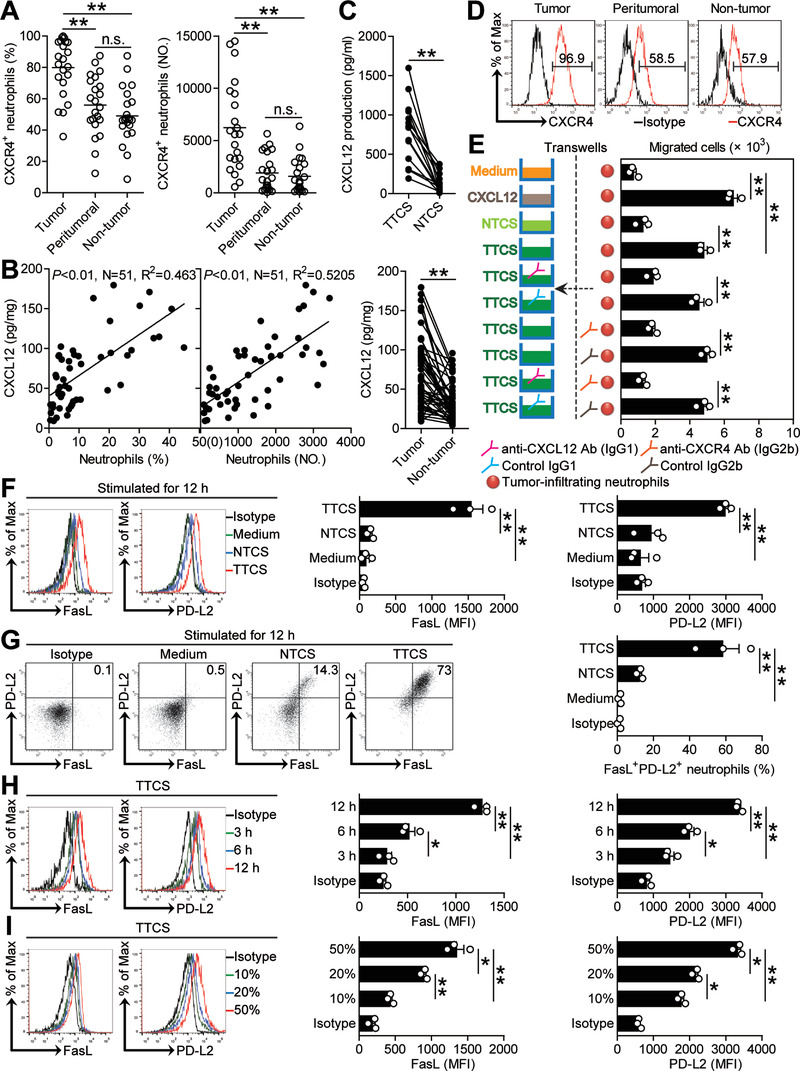
Human GC environments contribute to neutrophil infiltration and induce FasL^+^PD‐L2^+^ neutrophil subset. A) Statistics analysis of CXCR4^+^ neutrophil percentage in total neutrophils and the number of CXCR4^+^ neutrophils per million total cells in each samples of patients with GC by gating on CD45^+^CD11b^+^CD66b^+^CD15^+^CXCR4^+^ cells and counting (*n* = 23). B) The correlations between neutrophils and CXCL12 in GC tumors were analyzed. C) CXCL12 concentration between autologous tumor and non‐tumor tissues (*n* = 51) or between autologous TTCS and NTCS (*n* = 14) was analyzed. D) Expression of molecule CXCR4 on neutrophils. Color histograms represent staining of CXCR4. E) Migration of tumor‐infiltrating neutrophils was assessed by Transwell assay as described in the Experimental Section and statistically analyzed (*n* = 3). F) Representative data and statistical analysis of the expression of FasL and PD‐L2 on neutrophils exposed to 50% autologous TTCS and NTCS for 12 h. G) Representative data and statistical analysis of the induction of FasL^+^PD‐L2^+^ neutrophils exposed to 50% autologous TTCS and NTCS for 12 h. H) Representative data and statistical analysis of the expression of FasL and PD‐L2 on neutrophils exposed to 50% TTCS for 3, 6, and 12 h. I) Representative data and statistical analysis of the expression of FasL and PD‐L2 on neutrophils exposed to 10%, 20%, and 50% TTCS for 12 h. Data are mean ± SEM and analyzed by Student's *t*‐test, Mann‐Whitney U‐test, and one‐way ANOVA. *P < 0.05, **P < 0.01 for groups connected by horizontal lines. MFI: mean fluorescence intensity.

The results described above suggested that GC environments might trigger the accumulation of neutrophils and subsequently induce FasL^+^PD‐L2^+^ neutrophil subset. We therefore first investigated neutrophil chemotactic factors and found that tumor‐infiltrating neutrophils expressed higher CXCR4 than that on peritumoral or non‐tumor neutrophils (**Figure** **3**A,D). We further demonstrated that increased neutrophils, both in percentage and absolute number, positively correlated with increased CXCL12, the ligand for CXCR4, in GC tumors (Figure 3B), and that the concentrations of CXCL12 in tumor tissues or tumor tissue culture supernatants (TTCS) were significantly increased when compared to those in non‐tumor tissues or non‐tumor tissue culture supernatants (NTCS) (Figure 3C). Similar observations were made when analyzing the expressions of CXCL12 (Figure S5, Supporting Information). To substantiate the functional significance of CXCL12‐CXCR4 in the recruitment of neutrophils, tumor‐infiltrating neutrophils were isolated and neutrophil chemotaxis assays were performed. The results showed that TTCS induced significantly more tumor‐infiltrating neutrophils to migrate than NTCS from the same GC patients, and such migration was blocked upon pre‐treatment with neutralizing antibodies against CXCL12 and/or CXCR4 (Figure 3E). Taken together, these data support a model wherein GC tumors secrete chemokine CXCL12 to recruit neutrophils into the tumor environment via the CXCL12‐CXCR4 interaction.

Meanwhile, we hypothesized that GC environments might contribute to the induction of FasL^+^PD‐L2^+^ neutrophil subset. Consistent with our hypothesis, we stimulated neutrophils with NTCS or TTCS from autologous tumor or non‐tumor tissues, and found that, compared to NTCS‐conditioned neutrophils, TTCS‐conditioned neutrophils significantly upregulated both FasL and PD‐L2 expression (Figure 3F) and were induced to more FasL^+^PD‐L2^+^ neutrophil subset (Figure 3G). And this upregulation of FasL and PD‐L2 expression was induced in both time‐dependent (Figure 3H) and dose‐dependent (Figure 3I) manners. These findings together imply that GC environments contribute to neutrophil infiltration and induce FasL^+^PD‐L2^+^ neutrophil subset.

### 2.4 Th17 Cell‐Derived IL‐17A Induces Neutrophil FasL Expression via Activating ERK‐NF‐*κ*B Signaling Pathway

**Figure 4:**
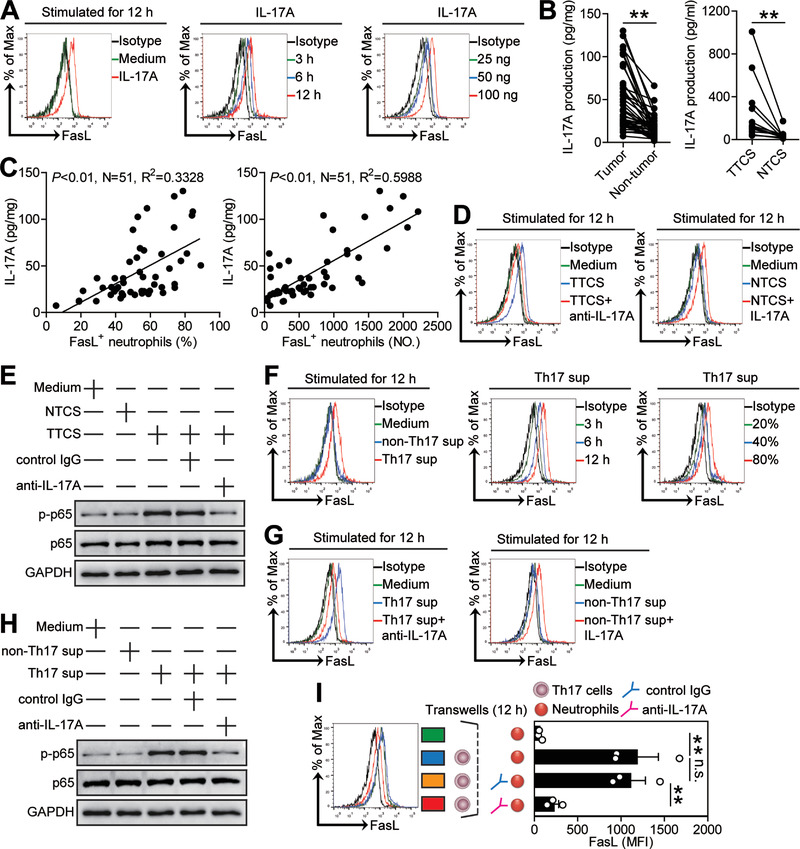
Th17 cell‐derived IL‐17A induces neutrophil FasL expression via activating ERK‐NF‐*κ*B signaling pathway. A) Expression of FasL on neutrophils exposed to IL‐17A (100 ng mL^−1^) or medium control for 12 h, or exposed to IL‐17A (100 ng mL^−1^) for 3, 6, and 12 h, or exposed to IL‐17A (25, 50, or 100 ng mL^−1^) for 12 h. B) IL‐17A concentration between autologous tumor and non‐tumor tissues (*n* = 51) or between autologous TTCS and NTCS (*n* = 14) was analyzed. C) The correlations between IL‐17A and FasL^+^ neutrophils in human tumors were analyzed. D) Expression of FasL on neutrophils exposed to TTCS with anti‐IL‐17A antibody or NTCS with IL‐17A for 12 h. E) The p65 and p‐p65 proteins in neutrophils exposed to autologous TTCS, NTCS, or TTCS with anti‐IL‐17A antibody or control IgG for 12 h were analyzed by western blot. F) Expression of FasL on neutrophils exposed to Th17 sup, non‐Th17 sup, or medium control for 12 h, or exposed to Th17 sup for 3, 6, and 12 h, or exposed to Th17 sup (20%, 40%, or 80%) for 12 h. G) Expression of FasL on neutrophils exposed to Th17 sup with anti‐IL‐17A antibody or non‐Th17 sup with IL‐17A for 12 h. H) The p65 and p‐p65 proteins in neutrophils exposed to Th17 sup, non‐Th17 sup, or Th17 sup with anti‐IL‐17A antibody or control IgG for 12 h were analyzed by western blot. I) FasL induction was assessed by Transwell assay as described in the Experimental Section and statistically analyzed (*n* = 3). Data are mean ± SEM and analyzed by Student's *t*‐test, Mann‐Whitney U‐test, and one‐way ANOVA. *P < 0.05, **P < 0.01 for groups connected by horizontal lines. Th17 sup: Th17 cell culture supernatants; non‐Th17 sup: non‐Th17 cell culture supernatants.

Tumor environment can possess various soluble factors, including proinflammatory cytokines. To see which cytokines might induce FasL on neutrophils, we first screened proinflammatory cytokines in human GC environments by microarray (Figure S6A, Supporting Information), and stimulated neutrophils with highly expressed cytokines including G‐CSF, M‐CSF, GM‐CSF, TGF‐*β*, IL‐1*β*, IL‐4, IL‐6, IL‐10, IL‐12, IL‐17A, IL‐17F, IL‐21, IL‐22, IL‐23, and IL‐33. We found that only IL‐17A upregulated the expression of FasL on neutrophils in both time‐ and dose‐dependent manners (**Figure** **4**A; Figure S6B,C, Supporting Information). Next, we found that the concentrations of IL‐17A in tumor tissues or TTSC were significantly increased when compared to that in non‐tumor tissues or NTCS (Figure 4B) and that there was clearly a positive correlation between IL‐17A production and FasL^+^ neutrophil infiltration within tumors (Figure 4C). Similar observations were made when analyzing the expressions of IL‐17A (Figure S6D,E, Supporting Information). Interestingly, blockade of IL‐17A in TTCS/neutrophil co‐culture efficiently inhibited the induction of FasL on neutrophils (Figure 4D; Figure S7A, Supporting Information); provision of exogenous IL‐17A into NTCS/neutrophil co‐culture significantly increased FasL expression on neutrophils (Figure 4D; Figure S7A, Supporting Information). These findings show that tumor‐derived IL‐17A plays an essential role in neutrophil FasL induction.

Signaling pathway inhibition experiments showed that only blocking the signal transduction of ERK with inhibitor U0126 or blocking the signal transduction of NF‐*κ*B with inhibitor BAY 11‐7082 effectively suppressed FasL expression on TTCS‐conditioned neutrophils or IL‐17A‐stimulated neutrophils either alone or in combination (Figure S7B–D, Supporting Information). Furthermore, p65, a direct NF‐*κ*B pathway downstream substrate, was predominantly phosphorylated in neutrophils after treatment with TTCS, and this phosphorylation was abolished when blocking IL‐17A (Figure 4E), implying that activation of ERK‐NF‐*κ*B signaling pathway is crucial for neutrophil FasL induction in GC environments.

Given the superior ability of Th17 cells to produce IL‐17A, we polarized Th17 cells in vitro, and stimulated neutrophils with Th17 cell culture supernatants, and found that these supernatants were superior to non‐Th17 cell culture supernatants in inducting neutrophil FasL expression in both time‐ and dose‐dependent manners (Figure 4F; Figure S8A, Supporting Information) via activation of ERK‐NF‐*κ*B signaling pathway (Figure S8B,C, Supporting Information). Furthermore, blockade of IL‐17A in Th17 cell culture supernatant/neutrophil co‐culture efficiently inhibited the induction of FasL on neutrophils; provision of exogenous IL‐17A into non‐Th17 cell culture supernatant/neutrophil co‐culture significantly increased FasL expression on neutrophils (Figure 4G; Figure S8D, Supporting Information); p65 was predominantly phosphorylated in neutrophils after treatment with Th17 cell culture supernatants, and this phosphorylation was abolished when blocking IL‐17A (Figure 4H).

The co‐localization of IL‐17A^+^ cells and CD15^+^ neutrophils (Figure S8E, Supporting Information) in the tumoral area of GC tissues suggested that Th17 cells might promote nearby neutrophils via IL‐17A. Therefore, we performed transwell assays and found that Th17 cell‐secreted IL‐17A was necessary for the induction of FasL expression on neutrophils (Figure 4I). These findings altogether show that Th17 cells secrete IL‐17A to induce neutrophil FasL expression by activating ERK‐NF‐*κ*B signaling pathway in GC environments.

### 2.5 Tumor Cell‐Derived G‐CSF Induces Neutrophil PD‐L2 Expression via Activating JAK‐STAT3 Signaling Pathway

**Figure 5:**
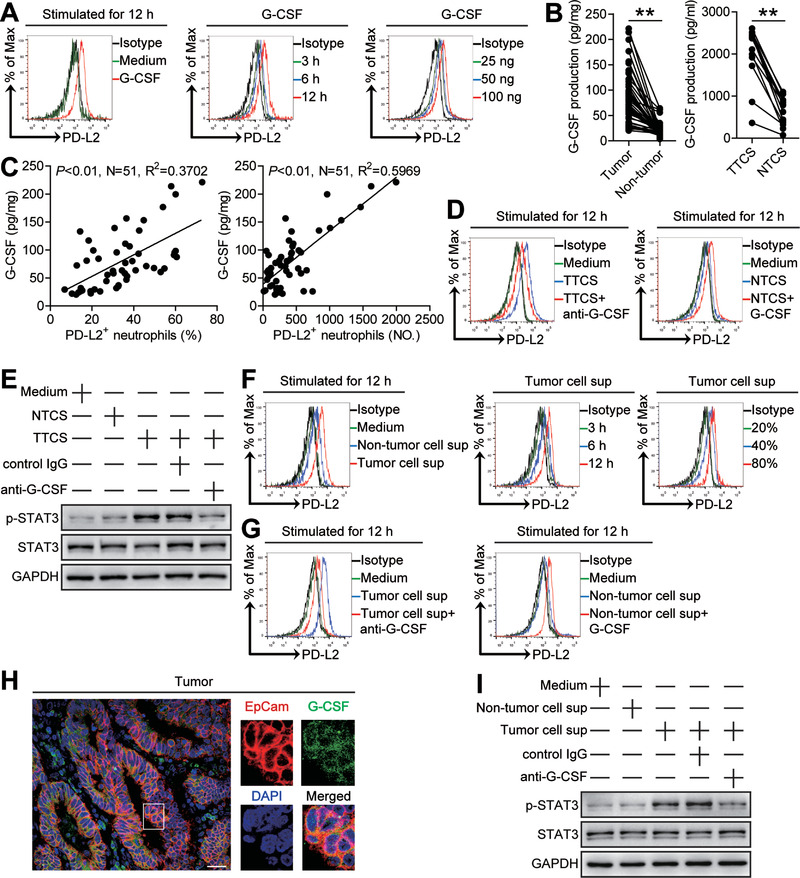
Tumor cell‐derived G‐CSF induces neutrophil PD‐L2 expression via activating JAK‐STAT3 signaling pathway. A) Expression of PD‐L2 on neutrophils exposed to G‐CSF (100 ng mL^−1^) or medium control for 12 h, or exposed to G‐CSF (100 ng mL^−1^) for 3, 6, and 12 h, or exposed to G‐CSF (25, 50, or 100 ng mL^−1^) for 12 h. B) G‐CSF concentration between autologous tumor and non‐tumor tissues (*n* = 51) or between autologous TTCS and NTCS (*n* = 14) was analyzed. C) The correlations between G‐CSF and PD‐L2^+^ neutrophils in human tumors were analyzed. D) Expression of PD‐L2 on neutrophils exposed to TTCS with anti‐G‐CSF antibody or NTCS with G‐CSF for 12 h. E) The STAT3 and p‐STAT3 proteins in neutrophils exposed to autologous TTCS, NTCS, or TTCS with anti‐G‐CSF antibody or control IgG for 12 h were analyzed by western blot. F) Expression of PD‐L2 on neutrophils exposed to tumor cell sup, non‐tumor cell sup or medium control for 12 h, or exposed to tumor cell sup for 3, 6, and 12 h, or exposed to tumor cell sup (20%, 40%, or 80%) for 12 h. G) Expression of PD‐L2 on neutrophils exposed to tumor cell sup with anti‐G‐CSF antibody or non‐tumor cell sup with G‐CSF for 12 h. H) Representative image of EpCam^+^G‐CSF^+^ cells in tumor tissues of GC patients by immunofluorescence staining. Scale bars: 50 µm. I) The STAT3 and p‐STAT3 proteins in neutrophils exposed to tumor cell sup, non‐tumor cell sup, or tumor cell sup with anti‐G‐CSF antibody or control IgG for 12 h were analyzed by western blot. Data are mean ± SEM and analyzed by Student's *t*‐test, Mann‐Whitney U‐test, and one‐way ANOVA. *P < 0.05, **P < 0.01 for groups connected by horizontal lines. Tumor cell sup: tumor cell culture supernatants; Non‐tumor cell sup: non‐tumor cell culture supernatants.

Next, to see which cytokines might induce PD‐L2 on neutrophils, we also stimulated neutrophils with highly‐expressed cytokines above, and found that only G‐CSF up‐regulated the expression of PD‐L2 on neutrophils in both time‐ and dose‐dependent manners (**Figure** **5**A; Figure S9A,B, Supporting Information). Next, we found that the concentrations of G‐CSF in tumor tissues or TTSC were significantly increased when compared to that in non‐tumor tissues or NTCS (Figure 5B), and that there was clearly a positive correlation between G‐CSF production and PD‐L2^+^ neutrophil infiltration within tumors (Figure 5C). Similar observations were made when analyzing the expressions of G‐CSF (Figure S9C,D, Supporting Information). Interestingly, blockade of G‐CSF in TTCS/neutrophil co‐culture efficiently inhibited the induction of PD‐L2 on neutrophils (Figure 5D; Figure S10A, Supporting Information); provision of exogenous G‐CSF into NTCS/neutrophil co‐culture significantly increased PD‐L2 expression on neutrophils (Figure 5D; Figure S10A, Supporting Information). These findings show that tumor‐derived G‐CSF plays an essential role in neutrophil PD‐L2 induction.

Signaling pathway inhibition experiments showed that only blocking the signal transduction of JAK with inhibitor AG490 and/or abolishing the phosphorylation of STAT3 with inhibitor FLLL32 effectively suppressed PD‐L2 expression on TTCS‐conditioned or G‐CSF‐stimulated neutrophils either alone or in combination (Figure S10B–D, Supporting Information). Furthermore, STAT3, a direct JAK‐STAT3 pathway downstream substrate, was predominantly phosphorylated in neutrophils after treatment with TTCS, and this phosphorylation was abolished when G‐CSF was blocked (Figure 5E), implying that activation of JAK‐STAT3 signaling pathway is crucial for neutrophil PD‐L2 induction in GC environments.

As EpCam^+^ tumor cells expressed G‐CSF in GC tumors (Figure 5H), we isolated EpCam^+^ cells from tumor and non‐tumor tissues of autologous GC patients, and cultured them to obtain tumor cell culture supernatants and non‐tumor cell culture supernatants, then stimulated neutrophils with these supernatants. We found that tumor cell culture supernatants were superior to non‐tumor cell culture supernatants in inducting neutrophil PD‐L2 expression in both time‐ and dose‐dependent manners (Figure 5F; Figure S11A, Supporting Information) via activation of ERK‐NF‐*κ*B signaling pathway (Figure S11B,C, Supporting Information). Furthermore, blockade of G‐CSF in tumor cell culture supernatant/neutrophil co‐culture efficiently inhibited the induction of PD‐L2 on neutrophils; provision of exogenous G‐CSF into non‐tumor cell culture supernatant/neutrophil co‐culture significantly increased PD‐L2 expression on neutrophils (Figure 5G; Figure S11D, Supporting Information); STAT3 was predominantly phosphorylated in neutrophils after treatment with tumor cell culture supernatants, and this phosphorylation was abolished when blocking G‐CSF (Figure 5I). Moreover, blockade of G‐CSF in TTCS/neutrophil co‐culture has no effects on the induction of FasL on neutrophils (Figure S11E, Supporting Information), and blockade of IL‐17A in TTCS/neutrophil co‐culture has no effects on the induction of PD‐L2 on neutrophils (Figure S11F, Supporting Information). These findings altogether show that G‐CSF from tumor cells induces neutrophil PD‐L2 expression by activating JAK‐STAT3 signaling pathway in GC environment.

### 2.6 Tumor‐Infiltrating and Tumor‐Conditioned Neutrophils Suppress CD8^+^ T‐Cell Immunity through FasL and PD‐L2

**Figure 6:**
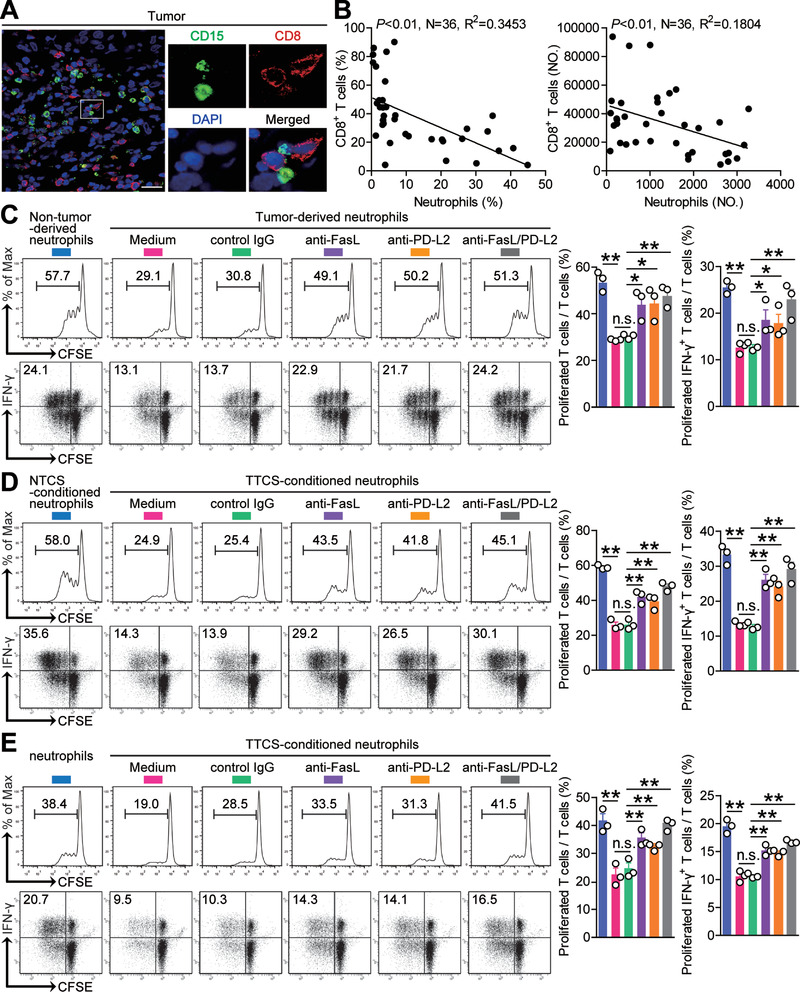
Tumor‐infiltrating and tumor‐conditioned neutrophils suppress CD8^+^ T‐cell immunity through FasL and PD‐L2. A) Representative image of CD15^+^ neutrophil (green) and CD8^+^ T cell (red) interactions in tumor tissues of GC patients by immunofluorescence. Scale bars: 20 µm. B) The correlations between neutrophils and CD8^+^ T cells in human GC tumors were analyzed. C) CFSE‐labeled peripheral CD8^+^ T cells of GC patients were co‐cultured for 5 d with autologous neutrophils from non‐tumor or tumor tissues with or without anti‐FasL and/or anti‐PD‐L2 antibody. Representative data and statistical analysis of T cell proliferation and proliferated IFN‐*γ*‐producing T cells were shown (*n* = 3). D) CFSE‐labeled peripheral CD8^+^ T cells of donors were co‐cultured for 5 d with autologous NTCS‐conditioned neutrophils or TTCS‐conditioned neutrophils with or without anti‐FasL and/or anti‐PD‐L2 antibody. Representative data and statistical analysis of T cell proliferation and proliferated IFN‐*γ*‐producing T cells were shown (*n* = 3). E) CFSE‐labeled tumor‐specific CD8^+^ T cells of donors were co‐cultured for 5 d with autologous normal neutrophils or TTCS‐conditioned neutrophils with or without anti‐FasL and/or anti‐PD‐L2 antibody. Representative data and statistical analysis of T cell proliferation and proliferated IFN‐*γ*‐producing T cells were shown (*n* = 3). Data are mean ± SEM and analyzed by Student's *t*‐test, Mann‐Whitney U‐test, and one‐way ANOVA. *P < 0.05, **P < 0.01, n.s. *P* > 0.05 for groups connected by horizontal lines.

The co‐localization of neutrophils and CD8^+^ T cells in the tumoral area of GC tissues (**Figure** **6**A) and the significant negative correlations between the levels of neutrophils and CD8^+^ T cells in GC tumors analyzed (Figure 6B) suggests that these neutrophils may promote tumor progression by impairing CD8^+^ T‐cell immunity. Neutrophils from tumor and non‐tumor tissues of autologous GC patients were therefore isolated and cultured with purified autologous peripheral blood CD8^+^ T cells for 5 d. Neutrophil/T‐cell co‐cultures showed that tumor‐infiltrating neutrophils were superior to non‐tumor‐derived neutrophils in inhibiting T cell proliferation and IFN‐*γ* production, which could be significantly attenuated by blockade of FasL and/or PD‐L2 (Figure 6C), suggesting an immunosuppressive function of tumor‐infiltrating neutrophils in tumor immunity via FasL and PD‐L2.

As tumor‐infiltrating neutrophils inhibited CD8^+^ T cells to greater degree than non‐tumor neutrophils, we hypothesized that tumor environment itself might play important roles in this process. Purified peripheral CD8^+^ T cells were co‐cultured with TTCS‐ or NTCS‐conditioned autologous blood neutrophils for 5 d. Interestingly, TTCS‐conditioned neutrophils showed significantly more suppression on IFN‐*γ* production and T cell proliferation, which was efficiently attenuated by blocking FasL and/or PD‐L2 (Figure 6D).

To demonstrate such neutrophil suppressive effect more directly, we generated tumor‐specific CD8^+^ T cells and co‐cultured them with autologous normal or TTCS‐conditioned blood neutrophils as above. The results showed that TTCS‐conditioned neutrophils significantly suppressed the proliferation and IFN‐*γ* production of tumor‐specific CD8^+^ T cells in FasL‐ and PD‐L2‐ dependent manners (Figure 6E). These results indicate that, in GC environment, neutrophils acquire ability to suppress CD8^+^ T‐cell function through FasL and PD‐L2.

### 2.7 Blockade of Neutrophil‐Associated FasL and PD‐L2 on Tumor‐Specific CD8^+^ T‐Cell Immunity Inhibits Tumor Growth and GC Progression

**Figure 7:**
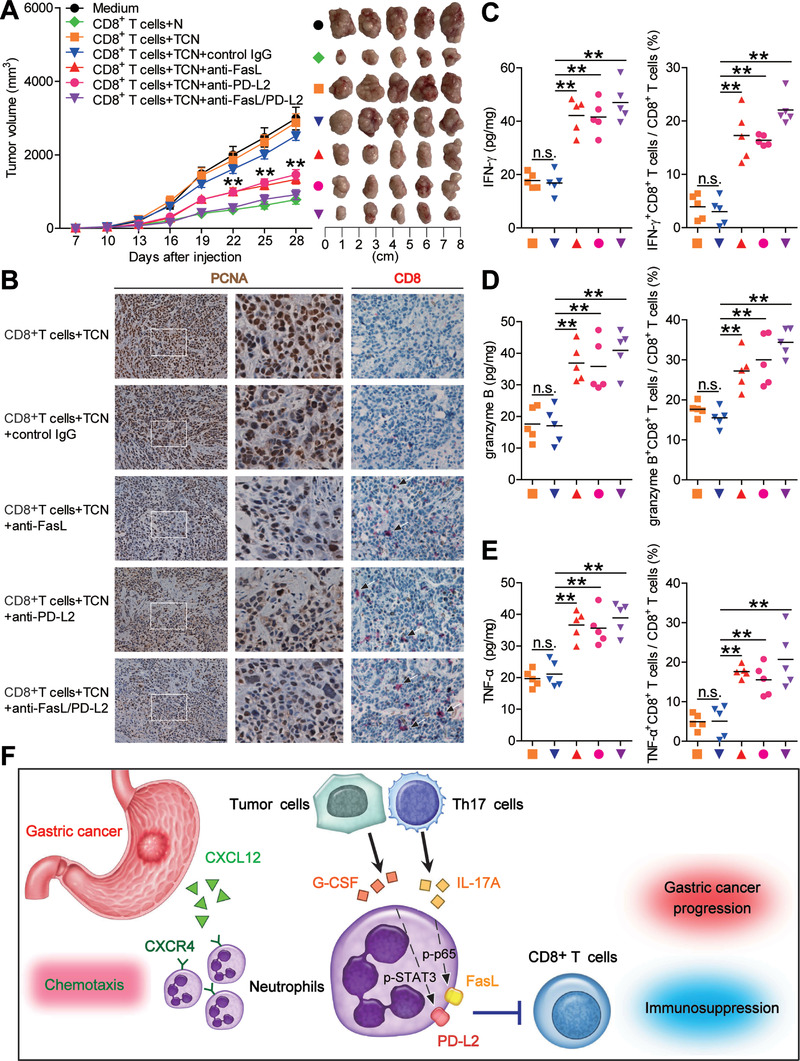
Blockade of neutrophil‐associated FasL and PD‐L2 on tumor‐specific CD8^+^ T‐cell immunity inhibits tumor growth and GC progression. A) Mice were injected with human SGC‐7901 cells, as described in the Experimental Section. The control animals received no further injections. The experimental treatments entailed injections with tumor‐specific CD8^+^ T cells in combination with untreated neutrophils (N) or TTCS‐conditioned neutrophils (TCN), or TCN pretreated with anti‐FasL and/or anti‐PD‐L2 antibody or a control IgG. The illustrated data represent tumor volumes (five mice in each group). The day of tumor cell injection was counted as day 0. **P < 0.01, for groups injections with TCN pretreated with anti‐FasL and/or anti‐PD‐L2 antibody, compared with groups injections with TCN pretreated with a control IgG. The tumors were excised and photographed 28 d after injecting the tumor cells. B) Proliferating cell nuclear antigen (PCNA) expression (brown) or CD8^+^ T cell infiltration (red) in tumors, C) IFN‐*γ* expression in tumors and IFN‐*γ*‐producing CD8^+^ T cell response in spleens, D) granzyme B expression in tumors and granzyme B‐producing CD8^+^ T cell response in spleens, and E) TNF‐*α* expression in tumors and TNF‐*α*‐producing CD8^+^ T cell response in spleens of mice injected with tumor‐specific CD8^+^ T cells in combination with TTCS‐conditioned neutrophils (TCN), or TCN pretreated with anti‐FasL and/or anti‐PD‐L2 antibody or a control IgG on day 28 after tumor cell injection were compared. F) A proposed model of cross‐talks among neutrophils, Th17 cells, tumor cells, and CD8^+^ T cells leading to FasL^+^PD‐L2^+^ neutrophil‐mediated immunosuppression and tumor progression in GC environment. Data are mean ± SEM and analyzed by Student's *t*‐test, Mann‐Whitney U‐test, and one‐way ANOVA. *P < 0.05, **P < 0.01, n.s. *P* > 0.05 for groups connected by horizontal lines.

To test the suppressive effect of FasL^+^PD‐L2^+^ neutrophils on tumor‐specific CD8^+^ T‐cell immunity in vivo, we treated TTCS‐conditioned neutrophils (TCN) with FasL and/or PD‐L2 blocking antibody or control IgG and then injected them together with autologous tumor‐specific CD8^+^ T cells into our established human NOD/SCID mice bearing SGC7901‐derived GC. As expected, mice without T cell transfer, or treated with T cells plus TCN or control IgG‐treated TCN showed tumor growth and disease progression (**Figure** **7**A; Figure S12A, Supporting Information). However, mice received with T cell transfer plus FasL and/or PD‐L2 blocking antibody‐treated TCN showed reduced tumor volumes and disease progression at each measurement time point from day 22 (Figure 7A), indicating a vital role of FasL^+^PD‐L2^+^ neutrophils in assisting tumors in vivo. Moreover, mice treated with T cells plus FasL and/or PD‐L2 blocking antibody‐treated TCN, also showed a decreased tumor growth with increased CD8^+^ T cell infiltration (Figure 7B; Figure S12B, Supporting Information), and increased IFN‐*γ* (Figure 7C; Figure S12C, Supporting Information), granzyme B (Figure 7D; Figure S12D, Supporting Information), and TNF‐*α* production (Figure 7E; Figure S12E, Supporting Information) in tumors or from spleen CD8^+^ T cells, compared with the mice treated with T cells plus TCN or control IgG‐treated TCN. Moreover, these untreated neutrophils did not express FasL (Figure S12F, Supporting Information), suggesting the use of FasL antibody would most likely have no effect on the untreated neutrophil group. Furthermore, blocking the CXCR4‐CXCL12 axis with CXCR4 and/or CXCL12 neutralizing antibodies did not augment the proliferation and IFN‐*γ* production of tumor‐specific CD8^+^ T cells co‐cultured with TCN (Figure S12G, Supporting Information), suggesting that targeting CXCR4/CXCL12 would most likely have no effect on the neutrophil suppressive function. These findings suggest that tumor‐associated neutrophils suppress tumor‐specific CD8^+^ T‐cell immunity in vivo through their surface FasL and PD‐L2 to promote tumor growth and GC progression.

## 3 Discussion

In this study, we have identified a novel protumorigenic FasL^+^PD‐L2^+^ neutrophil subset and have applied multiple complementary strategies to map the phenotype, mechanisms of induction, biologic function, and clinical relevance of these cells in the tumor environment of patients with GC. We show that within GC FasL^+^PD‐L2^+^ neutrophils play an active role on promoting tumor progression. Although neutrophils have already been described in patients with tumors,^[^ ^13^ ^]^ to our knowledge this is the first demonstration of a statistically significant correlation between prevalent high FasL^+^PD‐L2^+^ neutrophil subset in human tumors and poor patient prognosis; it is also the first demonstration for Th17 cell‐derived IL‐17A and tumor cell‐derived G‐CSF to collectively induce immunosuppressive neutrophils which inhibit tumor‐specific CD8^+^ T‐cell immunity. Our study therefore connects mechanistically the pathological role of neutrophils within the tumor environment.

Neutrophils have been found to be increased in GC tumors for many years. Here, we confirmed this phenomenon in our collected GC samples that increased infiltration of neutrophils in GC tissues was correlated with disease progress. Moreover, according to the difference of statistic data of neutrophils between peritumoral and non‐tumor tissues by flow cytometry or by immunohistochemistry, we think that, on one hand, it is likely that late stage (III+IV) patients have both specific and non‐specific neutrophil infiltration into the tumor and non‐tumor tissues as they have higher numbers in the circulation, and that, on the other hand, this may due to the difference of numbers of GC patients in different cohorts, suggesting further cohorts with more GC patients would be recruited to analyze neutrophils in GC tumors.

Very little is currently known about the subsets of tumor‐infiltrating neutrophils and their clinical relevance. Our profiling of neutrophils within GC confirms that tumor‐infiltrating neutrophils are phenotypically distinct from their peripheral counterparts that express little immunosuppressive molecule FasL and PD‐L2. Most interestingly, neutrophils co‐expressing FasL and PD‐L2 proteins can be induced in vitro simply by using tumor culture supernatants, and these FasL^+^PD‐L2^+^ neutrophils exhibit a unique phenotype in vivo, indicating that FasL^+^PD‐L2^+^ neutrophil subset can be generated under pathological tumor conditions and that their main role is likely direct immunosuppression.

Immunosuppression has been known as a hallmark of cancer, including various immune co‐inhibitory signals and cell death/apoptosis signals. The cross‐talks between PD‐1 and PD‐L1/PD‐L2^[^ ^9^, ^10^ ^]^ or Fas and FasL1^[^ ^11^, ^12^ ^]^ are ones of the main mechanisms contributing to such suppression or dysfunction applied to tumor‐specific CD8^+^ T cells. Intratumoral PD‐L2 expression in colorectal cancer,^[^ ^14^ ^]^ glioma,^[^ ^15^ ^]^ and prostate cancer,^[^ ^16^ ^]^ and intratumoral FasL expression in gastric cancer,^[^ ^17^ ^]^ breast cancer,^[^ ^18^ ^]^ and esophageal squamous‐cell carcinoma^[^ ^19^ ^]^ are commonly observed. However, no study showed neutrophil’ PD‐L2 and FasL upregulation in tumor tissues simultaneously in human malignancy. In human GC tumors, we now are the first to report the induced high co‐expression of FasL and PD‐L2 on infiltrating neutrophils, which further exhibit suppressive role on activated CD8^+^ T‐cell proliferation and IFN‐*γ* production, emphasizing the importance of FasL‐Fas and PD‐L2‐PD‐1 pathways in tumor‐related immunosuppression. Moreover, we found that this increased FasL^+^PD‐L2^+^ neutrophil subset in GC also expressed CD274 that have been reported to exert inhibition on T cells,^[^ ^20^ ^]^ indicating that FasL^+^PD‐L2^+^ neutrophils possess multiple immunosuppressive mechanisms. It is reported that neutrophils from healthy donors mediated tumor cell line (A549 cells, etc.) growth inhibitory effect through early cell cycle arrest and that treatment with an antagonist Fas receptor in A549 cells or knocking out of the Fas gene in A549 cells restored tumor cell cycle and lessened neutrophil anti‐tumor effect,^[^ ^21^ ^]^ which seems to be different from our results. The discrepancy is likely to the result of different cells used in these studies, and it could be speculated that, at different tumor progress stages, tumor‐infiltrating neutrophils might have different functions. During the initial stages of tumor, neutrophils might have a transient tumor‐alleviating role;^[^ ^22^ ^]^ however, as disease progresses, neutrophils could be influenced by tumor‐derived factors to acquire tumor‐promoting phenotype and function.

At present, FasL and PD‐L2 regulation of neutrophils in human cancer is largely unclear. It has been reported that survivin enhances FasL expression in human colon cancer cells.^[^ ^23^ ^]^ Others have found that, in esophageal squamous cell carcinoma, IL‐17A‐stimulated B cells gain more FasL expression,^[^ ^24^ ^]^ which resembles our data on neutrophil's FasL regulation by Th17 cell‐derived IL‐17A in GC. Recently, IFN‐*γ* was shown to orchestrate PD‐L2 up‐regulation on gastric tumor cells,^[^ ^25^ ^]^ we have now added G‐CSF onto that list as GC tumor cell‐derived G‐CSF effectively induces PD‐L2 expression on neutrophils. Both IL‐17A and G‐CSF are pro‐inflammatory cytokines that regulate immune response.^[^ ^26^, ^27^ ^]^ The other gastrointestinal IL‐17A‐expressing and G‐CSF‐secreting tumors, including hepatocellular carcinoma^[^ ^28^, ^29^ ^]^ and pancreatic ductal adenocarcinomas,^[^ ^30^, ^31^ ^]^ albeit not very common, are among the most rapidly advancing ones due to a pro‐inflammatory cytokine‐mediated immunosuppression.^[^ ^32^ ^]^ Here, we identify IL‐17A and G‐CSF as novel pro‐inflammatory factors derived from Th17 cells and tumor cells that effectively induce FasL and PD‐L2 expression on one population of neutrophils via activation of ERK‐NF‐*κ*B and JAK‐STAT3 signaling pathways.

Tumors develop strategies to interfere with effective anti‐tumor immune responses, ranging from the induction or generation of suppressive immune populations to the deletion or functional impairment of tumor‐reactive T cells.^[^ ^33^, ^34^ ^]^ Our study identifies a novel protumorigenic FasL^+^PD‐L2^+^ neutrophil subset in GC and reveals that FasL^+^PD‐L2^+^ neutrophil subset contributes to tumor progression, which is consistent with our observations that advanced tumor stages are associated with significant increase of FasL^+^PD‐L2^+^ neutrophils in GC tumors. Thus, our study provides new insights for human cancer immunosuppression and anticancer therapies targeting these pathogenic cells.

## 4 Conclusion

In our case, we have identified a definitive mechanism covering above events, which collectively propose a model involving the progressive immunosuppression within GC (Figure 7F): First, CXCL12‐CXCR4 chemotaxis mediates the recruitment of neutrophils into GC environment. Second, IL‐17A and G‐CSF with pro‐inflammatory feature are produced by Th17 cells and tumor cells within GC environment. Third, released IL‐17A and G‐CSF induces the activation of ERK‐NF‐*κ*B and JAK‐STAT3 signaling pathways in neutrophils, a process that is accompanied by the induction of FasL and PD‐L2 expression on these cells. Finally, these immunosuppressive FasL^+^PD‐L2^+^ neutrophils exert protumorigenic effects by suppressing tumor‐specific CD8^+^ T‐cell immunity in FasL/PD‐L2 dependent manners. In the future, therapeutics aimed at interfering these pathological FasL^+^PD‐L2^+^ neutrophils may be developed to provide novel strategies for GC treatment.

## 5 Experimental Section

### Patients and Specimens

Samples were obtained from patients who underwent surgical resection at the Southwest Hospital of Third Military Medical University. None of the patients had received chemotherapy or radiation before the sample was taken. Patients with infectious diseases, autoimmune diseases, or multiple primary cancers were excluded. Fresh gastric tumor (homogeneous cellularity, without foci of necrosis), peritumoral and non‐tumor (non‐tumor tissues, at least 5 cm distant from the tumor site) tissues and autologous peripheral blood were obtained from 51 patients with GC who underwent surgical resections between July 2018 and August 2020 were used for flow cytometry analysis (Cohort 1, their clinical characteristics are described in Table S4, Supporting Information). Paraffin‐embedded or frozen tissues from 125 patients who underwent surgical resections between September 2012 and November 2015 were used for immunohistochemistry and immunofluorescence analysis (Cohort 2, their clinical characteristics are described in Table S5, Supporting Information). The clinical stages of the tumors were determined according to the TNM classification system of the International Union against Cancer (8th ed.). The study was approved by the Ethics Committee of the Southwest Hospital of Third Military Medical University. Each subject provided written informed consent. Antibodies and other reagents are listed in Table S6 (Supporting Information). A schematic diagram that recalled the entire strategy is shown in Figure S13 (Supporting Information).

### Isolation of Single Cells from GC Tissues

Fresh tissues were washed three times with Hank's solution containing 1% fetal calf serum (FCS) and cut into small pieces. Specimens were collected in complete RPMI 1640 medium supplemented with 10% FCS (R‐10) containing collagenase IV (1 mg mL^−1^) and deoxyribonuclease I (10 mg mL^−1^) and mechanically separated using the gentle MACS Dissociator (Miltenyi Biotec). Dissociated cell suspensions were further incubated for 1 h under continuous rotation at 37 °C. The cell suspensions were then filtered by a 70 µm cell filter (BD Labware). Cell viability, as measured by trypan blue exclusion staining, was typically >95%.

### Isolation of Neutrophils, Tumor Cells, Th17 Cells, and CD8^+^ T Cells

As mentioned above, single cells of tumor and non‐tumor tissues were prepared. Then the single cell suspension was stained with anti‐human CD45, anti‐human CD11b, anti‐human CD66b, and anti‐human CD15 antibodies, and neutrophils from autologous tumor and non‐tumor tissues were sorted by fluorescence‐activated cell sorter (FACS) (FACSAria III; BD Biosciences) by gating on CD45^+^CD11b^+^CD66b^+^CD15^+^ live cells. Density gradient centrifugation was used to isolate peripheral blood mononuclear cells (PBMCs) from autologous GC patients and healthy donors by using Ficoll‐Paque Plus. Blood neutrophils were harvested after lyses of red blood cells with lyses solution. CD8^+^ T cells from PBMCs were purified with using anti‐CD8 (StemCell Technologies) magnetic beads. For Th17 cells, CD4^+^ T cells from PBMCs were purified using anti‐CD4 (StemCell Technologies) magnetic beads, and then polarized in R‐10 containing human recombinant (hr) IL‐2 (20 IU mL^−1^), anti‐CD3 (2 µg mL^−1^), and anti‐CD28 (1 µg mL^−1^) antibodies, in the presence of hr IL‐1*β* (10 ng mL^−1^), IL‐6 (10 ng mL^−1^), and IL‐23 (10 ng mL^−1^). After 5‐d incubation, Th17 cells were purified using human IL‐17 Secretion Assay‐Detection Kits (Miltenyi Biotec), and the remaining cells were as non‐Th17 cells. Tumor cells and non‐tumor cells from single cells of autologous tumor and non‐tumor tissues were purified with using anti‐EpCam (Miltenyi Biotec) magnetic beads. The sorted cells were used only when their viability was determined >95% and their purity was determined >95%.

### Preparation of Tumor Cell or Non‐Tumor Cell Culture Supernatants and Th17 Cell or Non‐Th17 Cell Culture Supernatants

Tumor cell culture supernatants or non‐tumor cell culture supernatants were prepared by plating 5 × 10^6^ cells purified tumor cells and non‐tumor cells from autologous tumor and non‐tumor tissues in 1 mL R‐10 for 24 h. Th17 cell culture supernatants or non‐Th17 cell culture supernatants were prepared by plating 5 × 10^6^ cells purified Th17 cells and non‐Th17 cells from autologous donors in 1 mL R‐10 for 24 h. The supernatant was then centrifuged and harvested.

### Preparation of TTCS and NTCS and Supernatant‐Conditioned Neutrophils

TTCS or NTCS were prepared by plating autologous tumor or non‐tumor gastric tissues in 1 mL RPMI 1640 medium for 24 h. The supernatants were then harvested by centrifugation. To generate supernatant‐conditioned neutrophils, neutrophils were first harvested and cultured with 50% TTCS or NTCS for 12 h, and then washed with RPMI‐1640 medium for three times. Neutrophils cultured with RPMI‐1640 medium were used as controls.

### Chemotaxis Assay

FACS sorted tumor‐infiltrating neutrophils (1 × 10^5^) from fresh human tumor tissues were transferred into the upper chambers of 3‐µm pore size Transwells (Corning). Autologous 50% TTCS or NTCS as the sources of chemoattractants were placed in the lower chambers. After 30‐min culture at 37 °C, migration was quantified by counting cells in the lower chamber and cells adhering to the bottom of the membrane. In some cases, blocking antibody for CXCR4 (20 µg mL^−1^, IgG2b) or control IgG2b was added into neutrophil suspensions and incubated for 2 h before chemotaxis assay. Furthermore, CXCL12 neutralizing antibody (20 µg mL^−1^, IgG1) or control IgG1 was added into TTCS in some assays. RPMI‐1640 medium and chemokine CXCL12 (100 ng mL^−1^) were placed in the lower chambers as blank and positive controls, respectively.

### Neutrophil Stimulation

Neutrophils from healthy donors were stimulated with 50% TTCS or 50% autologous NTCS, Th17 cell or non‐Th17 cell culture supernatants, tumor cell or non‐tumor cell culture supernatants for 12 h, or with 50% TTCS, Th17 cell culture supernatants, tumor cell culture supernatants for 3, 6, or 12 h, or with different concentrations TTCS (10%, 20%, 50%), Th17 cell culture supernatants (20%, 40%, 80%), tumor cell culture supernatants (20%, 40%, 80%) for 12 h, or with 50% TTCS or Th17 cell culture supernatants together with neutralizing antibody against human IL‐17A (20 µg mL^−1^), 50% autologous NTCS or non‐Th17 cell culture supernatants together with hr IL‐17A (100 ng mL^−1^) for 12 h, or with 50% TTCS or tumor cell culture supernatants together with neutralizing antibody against human G‐CSF (20 µg mL^−1^), 50% autologous NTCS or non‐tumor cell culture supernatants together with hr G‐CSF (100 ng mL^−1^) for 12 h, or with hr cytokines (100 ng mL^−1^) for 12 h. In some cases, neutrophils from healthy donors were cultured in the lower chambers of Transwells (0.4‐µm pore size) with autologous Th17 cells in the upper chambers of Transwells together with IL‐17A neutralizing antibody or control IgG (20 µg mL^−1^) for 12 h. After stimulation, the cells were harvested for flow cytometric analysis and western blot. For the signaling pathway inhibition experiments, the cells were pretreated with BAY 11‐7082 (an I*κ*B*α* inhibitor), U0126 (an ERK inhibitor), AG490 (a JAK inhibitor), FLLL32 (an STAT3 inhibitor), SP600125 (a c‐Jun N‐terminal kinase (JNK) inhibitor), SB203580 (a mitogen‐activated protein kinase (MAPK) inhibitor), Wortmannin (a PI3K inhibitor), or GSK‐3*β* inhibitor (10 × 10^−6^ m) for 1 h, then were stimulated with 50% TTCS, Th17 cell culture supernatants, tumor cell culture supernatants, IL‐17A or G‐CSF (100 ng mL^−1^) for 12 h and harvested as above. As the inhibitors were dissolved in dimethyl sulfoxide (DMSO), parallel cell groups were treated with DMSO or culture media as controls.

### Ex Vivo and In Vitro Neutrophil‐CD8^+^ T‐Cell Co‐Culture System

In an ex vivo co‐culture system, magnetic bead‐purified peripheral CD8^+^ T cells (2 × 10^5^ cells/well in 96‐well plates) were labeled with carboxyfluorescein succinimidyl ester (CFSE) and co‐cultured with autologous neutrophils isolated from tumor or non‐tumor tissues at a 2:1 (T‐cell:neutrophil) ratio in 200 µL (R‐10) containing rh IL‐2 (20 IU mL^−1^), anti‐CD3 (2 µg mL^−1^), and anti‐CD28 (1 µg mL^−1^) antibodies, with or without human FasL or PD‐L2 neutralizing antibody or control IgG (20 µg mL^−1^). In another in vitro co‐culture system, CFSE‐labeled magnetic bead‐purified peripheral CD8^+^ T cells (2 × 10^5^ cells/well in 96‐well plates) were co‐cultured with autologous TTCS‐ or NTCS‐conditioned neutrophils at a 2:1 ratio as described above, in the presence or absence of human FasL or PD‐L2 neutralizing antibody or control IgG (20 µg mL^−1^). After 5‐d incubation, the cells were harvested for intracellular cytokine staining.

### In Vitro Tumor‐Specific CD8^+^ T‐Cell Immunosuppression

Human GC cell line SGC‐7901 was purchased from China Center for Type Culture Collection (CCTCC, China, verified by using short tandem repeat profiling methods). Monocyte‐derived dendritic cells (DCs) from healthy donors were incubated with irradiated apoptotic SGC‐7901 cells at a 1:5 ratio for 24 h. Autologous magnetic bead‐purified peripheral CD8^+^ T cells (2 × 10^5^ cells/well in 96‐well plates) were activated by incubation with those tumor‐loaded DCs (2 × 10^4^ cells/well) in 200 µL R‐10 containing rh IL‐2 (20 IU mL^−1^), rh IL‐7 (10 ng mL^−1^), anti‐CD3 (2 µg mL^−1^), and anti‐CD28 (1 µg mL^−1^) antibodies for two weeks. Thereafter, a second stimulation was performed by further incubation with tumor‐loaded DCs for another two weeks to generate tumor‐specific CD8^+^ T cells. CFSE‐labeled tumor‐specific CD8^+^ T cells (2 × 10^5^ cells/well in 96‐well plates) were co‐cultured with autologous peripheral neutrophils, or TTCS‐conditioned neutrophils at a 2:1 ratio as described above, in the presence or absence of human FasL or PD‐L2 neutralizing antibody or control IgG (20 µg mL^−1^), or human CXCL12 neutralizing antibody or control IgG1 (20 µg mL^−1^), or human CXCL12 neutralizing antibody or control IgG2b (20 µg mL^−1^). After 5‐d incubation, the cells were harvested for intracellular cytokine staining.

### In Vivo Tumor Inhibition Assay

All animal experiments were undertaken with review and approval from the Animal Ethical and Experimental Committee of Third Military Medical University. A total of 10^6^ gastric cancer cells (SGC‐7901) in 100 µL of buffered saline were subcutaneously injected into the axillary tissues of female nonobese diabetic/severe combined immunodeficient (NOD/SCID) mice (for 5–7‐week, one tumor per mouse). Normal peripheral neutrophils from healthy donors were stimulated with 50% TTCS for 12 h. Then, 5 × 10^6^ tumor‐specific CD8^+^ T cells were co‐cultured with autologous peripheral neutrophils, or TTCS‐conditioned neutrophils at a 2:1 ratio in the presence or absence of human FasL and/or PD‐L2 neutralizing antibody or control IgG (20 µg mL^−1^) for 24 h as described above, and were subsequently injected into the peritoneum in 100 µL of buffered saline on day 10 after tumor inoculation. Tumor size was measured every 3 d by two independent observers using calipers fitted with a vernier scale. Tumor volume was calculated based on three perpendicular measurements. Once the mice were sacrificed, the tumors were photographed and weighed, and were further fixed for immunohistochemical staining, real‐time PCR, and ELISA, and the spleens were dissociated into single cells for flow cytometry.

### Flow Cytometry

Flow cytometric analysis was performed according to standard protocols. Cell surface markers were stained with specific or isotype control antibodies. For intracellular cytokine measurements, the cells were stimulated for 5 h with phorbol myristate acetate (50 ng mL^−1^) plus ionomycin (1 µg mL^−1^) in the presence of GolgiStop. Intracellular cytokine staining was performed after fixation and permeabilization using Perm/Wash solution. The cells were analyzed by multicolor flow cytometry with FACSCanto (BD Biosciences). Data were analyzed with Flowjo software (TreeStar) or FACSDiva software (BD Biosciences).

### Western Blot

Western blot assays were performed on 10% SDS‐PAGE gels using equivalent amounts of cell lysate proteins of samples. 5% BSA was used for blocking the PVDF membranes. Human p65, p‐p65, STAT3, and p‐STAT3 were detected with their antibodies, respectively. This was followed by incubation with horseradish peroxidase (HRP)‐conjugated secondary antibodies. Bound proteins were visualized by using SuperSignal West Dura Extended Duration Substrate kit.

### ELISA

Tissues were collected, homogenized in 1 mL sterile protein extraction reagent, and centrifuged. Tissue supernatants were collected for ELISA. Cell culture supernatants were collected as above for ELISA. Concentrations of IFN‐*γ*, granzyme B, and TNF‐*α* in the tumor tissues, concentrations of CXCL12, IL‐17A, and G‐CSF in the tumor or non‐tumor gastric tissues and TTCS or NTCS were determined using ELISA kits according to the manufacturer's instructions. For tissues, total protein was measured by using BCA Protein Assay Kit according to the manufacturer's instructions. The cytokine or chemokine concentrations in the tissues were expressed as picograms per milligrams of total protein.

### Immunohistochemistry

Paraformaldehyde‐fixed and paraffin‐embedded samples were cut into 5 µm sections. For immunohistochemical single‐staining, the sections were incubated with anti‐human CD15, anti‐human myeloperoxidase (MPO), anti‐human FasL, anti‐human PD‐L2, anti‐human IL‐17A, anti‐human CD8, and anti‐human proliferating cell nuclear antigen (PCNA) antibodies respectively, and then were stained by HRP‐conjugated anti‐mouse IgG/anti‐rabbit IgG or using EnVision G2 System/AP Rabbit/Mouse (Permanent Red) followed by diaminobenzidine. All the sections were finally counterstained with hematoxylin and examined using a microscope (Nikon Eclipse 80i; Nikon).

### Immunofluorescence

Paraformaldehyde‐fixed tumor tissue sections from GC patients were washed in PBS, blocked for 30 min with 20% goat serum in PBS, then stained for CD15 and CD8, or EpCam and G‐CSF. Slides were examined with a confocal fluorescence microscope (LSM 510 META, Zeiss).

### Microarray

Gene expression profiles of human tumor tissues from GC patients were analyzed with the Affymetrix GeneChip Human Gene 1.0 ST Array (Affymetrix), strictly following the manufacturer's protocol. Microarray experiments were performed at the Genminix Informatics (China) with the microarray service certified by Affymetrix.

### RNA Sequencing

Total RNA from FACS sorted tumor‐infiltrating FasL^+^PD‐L2^+^ neutrophils and autologous peripheral FasL^−^PD‐L2^−^ neutrophils of five GC patients was extracted using RNeasy Micro Kits (Qiagen, Germany), following the manufacturer's instructions. The concentration and quality of RNA samples were determined by the NanoDrop 2000 spectrophotometer (NanoDrop technologies, USA) and then used to construct strand‐specific (first cDNA strand) RNA libraries with VAHTS Stranded mRNA‐seq Library Prep Kit for Illumina v2 (Vazyme Biotech, China). RNA libraries for sequencing were performed on Illumina NovaSeq 6000 platform. Subsequently, 150 bp paired‐end reads were mapped to the reference human genome build hg38 by using HISAT2. The reads mapped the genome were calculated using HTSeq. Differential expression analysis presented here was performed with DESeq2 R package. Significantly differential expression genes (fold change ≥ 2 and *P* < 0.05) were used to generate the PPI network and visualized by Cytoscape software. GO and Kyoto Encyclopedia of Genes and Genomes (KEGG) pathway enrichment analyses were conducted by clusterProfiler R package. Hierarchically clustered heatmaps and volcano plots were visualized by pheatmap R package and ggplot2 R package.

### Real‐Time PCR

Extracted RNA from specimens was reverse‐transcribed to cDNA by PrimeScript RT reagent Kit. Real‐time PCR was performed on the IQ5 (Bio‐Rad) with the Real‐time PCR Master Mix according to the manufacturer's specifications. Expression of CXCL12, IL‐17A, G‐CSF, IFN‐*γ*, granzyme B, and TNF‐*α* in the tumor or non‐tumor tissues was measured using the SYBR green method with the respective primers (Table S7, Supporting Information). The relative gene expression was expressed as fold change calculated by the ΔΔCt method. Human GAPDH mRNA level served as a normalizer, and its level in non‐tumor tissues or in tumor tissues from the control animals bearing SGC7901‐derived GC served as a calibrator. The relative gene expression was expressed as fold change of relevant mRNA calculated by the ΔΔCt method, and the average level of gene expression in non‐tumor tissues or in tumor tissues from the control animals bearing SGC7901‐derived GC was defined as 1.

### Statistical Analysis

Results are expressed as mean ± SEM. Student's *t*‐test was generally used to analyze the differences between two groups, but when the variances differed, the Mann‐Whitney U‐test was used. Correlations between parameters were assessed using the Pearson correlation analysis and linear regression analysis as appropriate. Overall/disease‐free survival was defined as the interval between surgery and death/recurrence or between surgery and the last observation for surviving/disease‐free patients. The known tumor‐unrelated deaths (e.g., accidental death) were excluded from the death record for this study. Cumulative survival time was calculated by the Kaplan‐Meier method, and survival was measured in month; the log‐rank test was applied to compare between two groups. Multivariate analysis of prognostic factors for patient survival was performed using the Cox proportional hazards model. SPSS statistical software (version 13.0) was used for all statistical analysis. All data were analyzed using two‐tailed tests, and *P* < 0.05 was considered statistically significant.

### Study Approval

All breeding and experiments were undertaken with review and approval from the Animal Ethical and Experimental Committee of Third Military Medical University (2019YFC1302200). The experiments involving human samples were approved by the Ethics Committee of Southwest Hospital of Third Military Medical University (2018YFC1303300). The written informed consent was obtained from each subject.

## Conflict of Interest

The authors declare no conflict of interest.

## Author Contributions

Z.‐G.S., Y.‐L.Z., and J.‐Y.Z. contributed equally to this work. All listed authors participated meaningfully in the study, and they have seen and approved the submission of this manuscript. Y.Z. designed the research. Y.Z., Z.‐G.S., Y.‐L.Z., and J.‐Y.Z. participated in performing the research, analyzing the data, and initiating the original draft of the article. Y.Z., Z.‐G.S., Y.‐L.Z., J.‐Y.Z., and W.S.C. revised the manuscript. Z.‐G.S., Y.‐L.Z., Z.‐B.Y., T.‐T.W., F.‐Y.M., Y.‐S.T., L.‐S.P., W.‐Y.C., P.W., P.C., W.‐Q.T., and J.C. participated in performing the research and collecting the data. Y.‐L.Z., J.‐Y.Z., T.‐T.W., and J.C. contributed reagents and human clinical samples. Y.‐L.Z. and J.C. supervised the studies.

## Supporting information

Supporting Information

## Data Availability

The data that support the findings of this study are available from the corresponding author upon reasonable request.
