# Supplementary material for: FasL+PD‐L2+ Identifies a Novel Immunosuppressive Neutrophil Population in Human Gastric Cancer That Promotes Disease Progression
Source: Adv Sci (Weinh). 2021 Dec 26;9(5):2103543. doi: 10.1002/advs.202103543 (PMC8844550; doi:10.1002/advs.202103543)
Supplement: Supplementary file 1 — Supporting Information [file ADVS-9-2103543-s001.pdf]

ADVANCED SCIENCE
Open Access

Supporting Information

for *Adv. Sci.*, DOI: 10.1002/advs.202103543

FasL^{+}PD-L2^{+} Identifies a Novel Immunosuppressive Neutrophil Population in Human Gastric Cancer That Promotes Disease Progression

*Zhi-Guo Shan, Yong-Liang Zhao, Jin-Yu Zhang, Zong-Bao Yan, Ting-Ting Wang, Fang-Yuan Mao, Yong-Sheng Teng, Liu-Sheng Peng, Wan-Yan Chen, Pan Wang, Ping Cheng, Wen-Qing Tian, Jun Chen, Weisan Chen, and Yuan Zhuang**

Figure S1.

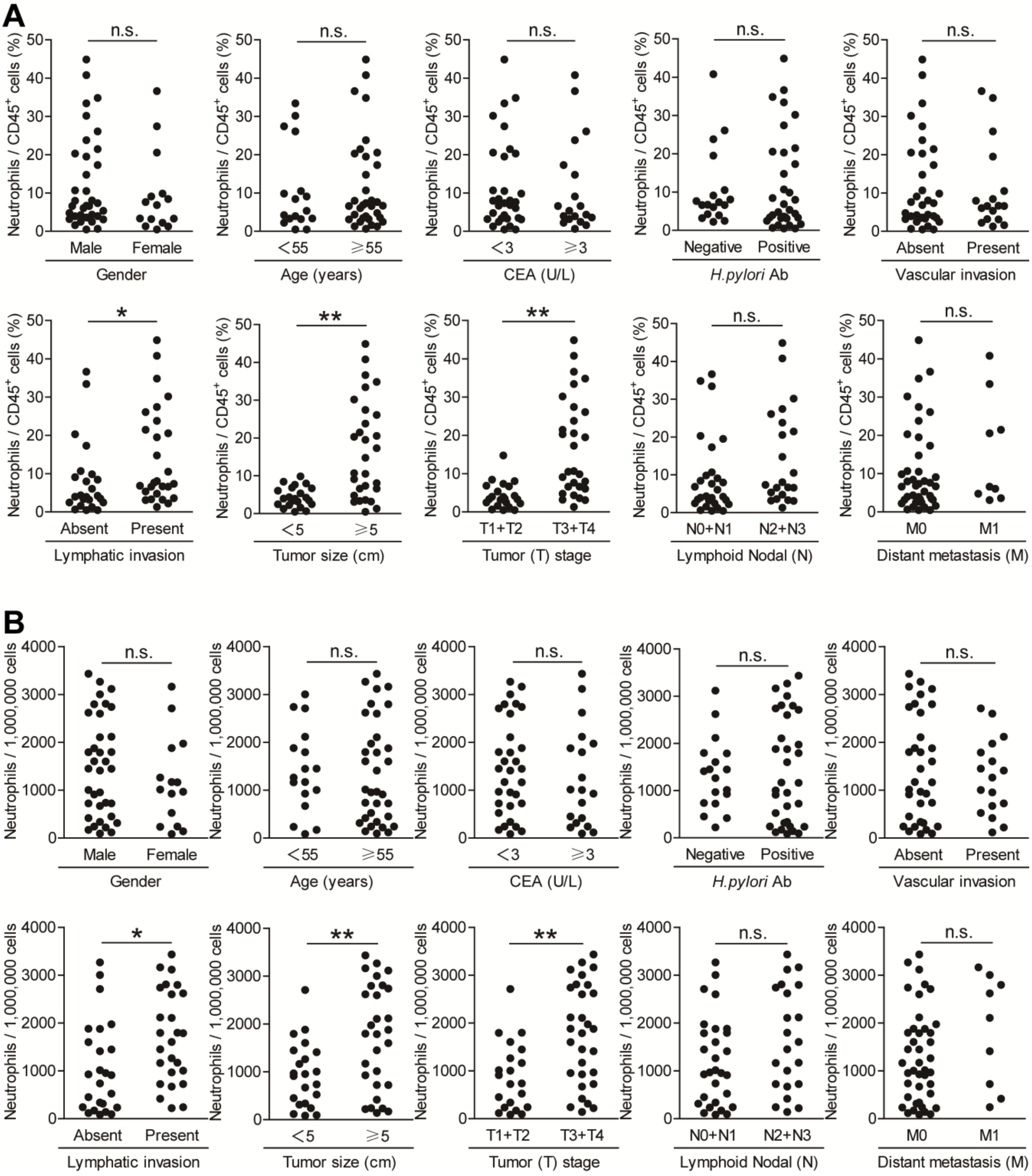

Figure S1. Neutrophil percentage or neutrophil number and its potential correlations with clinical parameters (cohort 1). (A and B) Neutrophil percentage in $CD45^+$ leukocytes (A) or neutrophil number per million total cells (B) was analyzed for correlations with clinical pathological parameters. Data are analyzed by Student *t* test and Mann-Whitney U test. **P*<0.05, ***P*<0.01, n.s *P*>0.05 for groups connected by horizontal lines. CEA, carcinoembryonic antigen; *H.pylori* Ab, *Helicobacter pylori* antibody.

Figure S2.

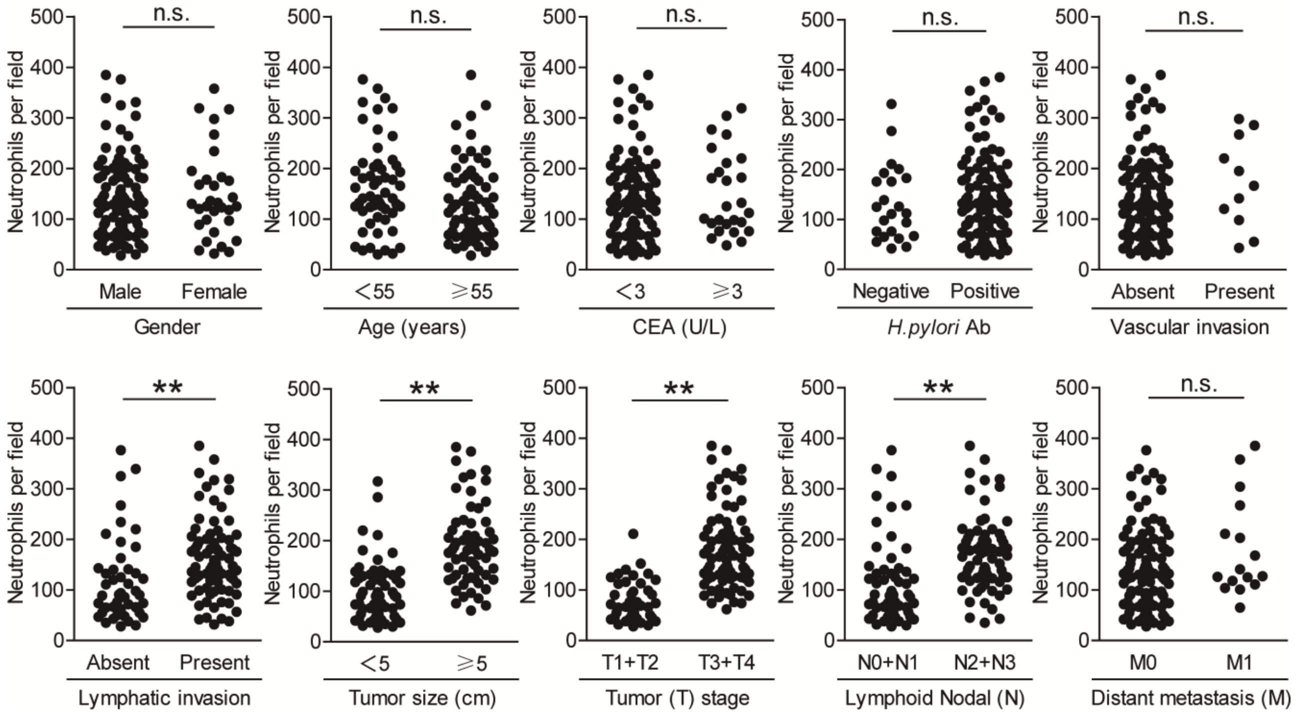

Figure S2. Neutrophil number and its potential correlations with clinical parameters (cohort 2). Neutrophil number per field was analyzed for correlations with clinical pathological parameters. Data are analyzed by Student *t* test and Mann-Whitney U test. **P*<0.05, ***P*<0.01, n.s *P*>0.05 for groups connected by horizontal lines. CEA, carcinoembryonic antigen; *H.pylori* Ab, *Helicobacter pylori* antibody.

Figure S3.

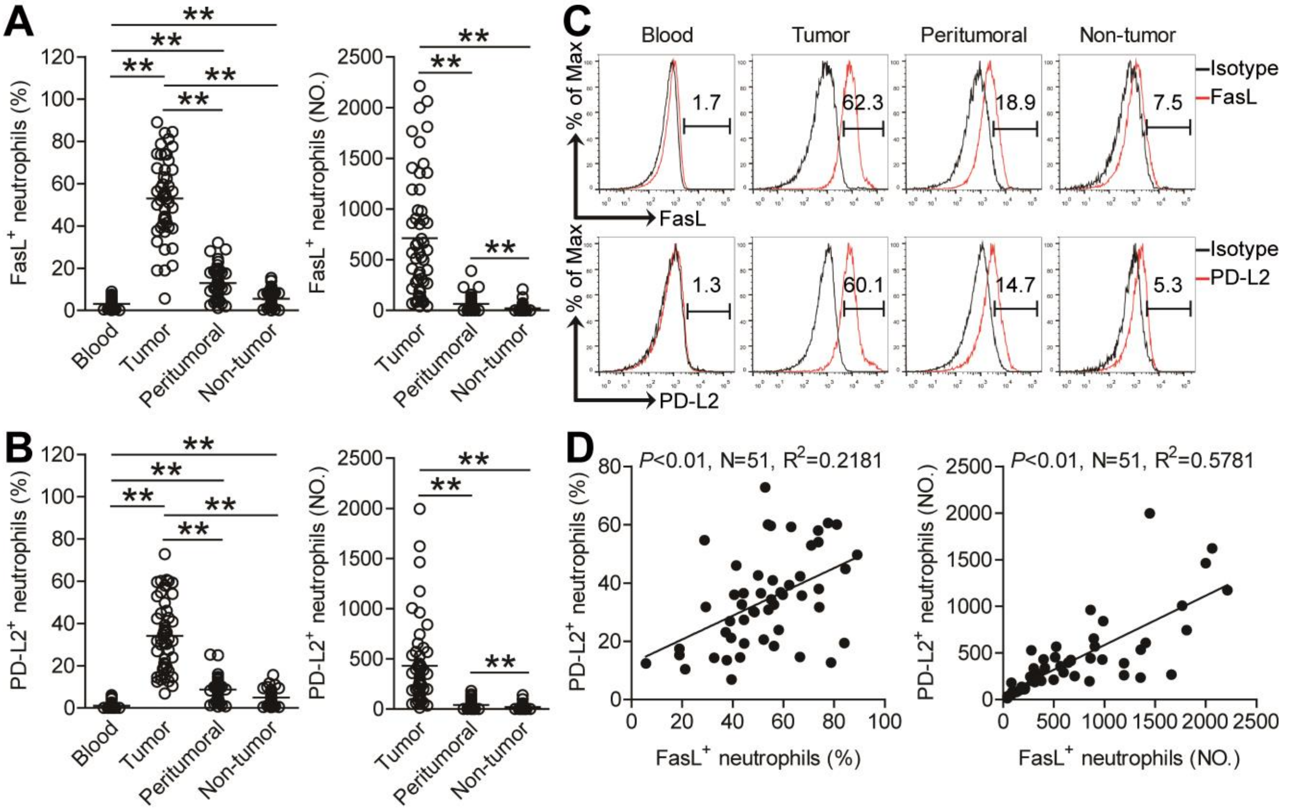

Figure S3. FasL$^+$PD-L2$^+$ neutrophil subset with a unique phenotype is increased in GC as tumor progresses. (A) Statistics analysis of FasL$^+$ neutrophil percentage or PD-L2$^+$ neutrophil percentage in total neutrophils in samples of patients with GC (n=51). (B) Statistics analysis of FasL$^+$ neutrophil number or PD-L2$^+$ neutrophil number per million total cells in samples of patients with GC (n=51). (C) Expression of molecules FasL and PD-L2 on neutrophils. Color histograms represent staining of functional molecule FasL and PD-L2. (D) The correlations between FasL$^+$ neutrophils and PD-L2$^+$ neutrophils in GC tumors were analyzed. Data are mean ± SEM and analyzed by Student *t* test, Mann-Whitney U test and 1-way ANOVA. *P<0.05, **P<0.01, n.s P>0.05 for groups connected by horizontal lines.

Figure S4.

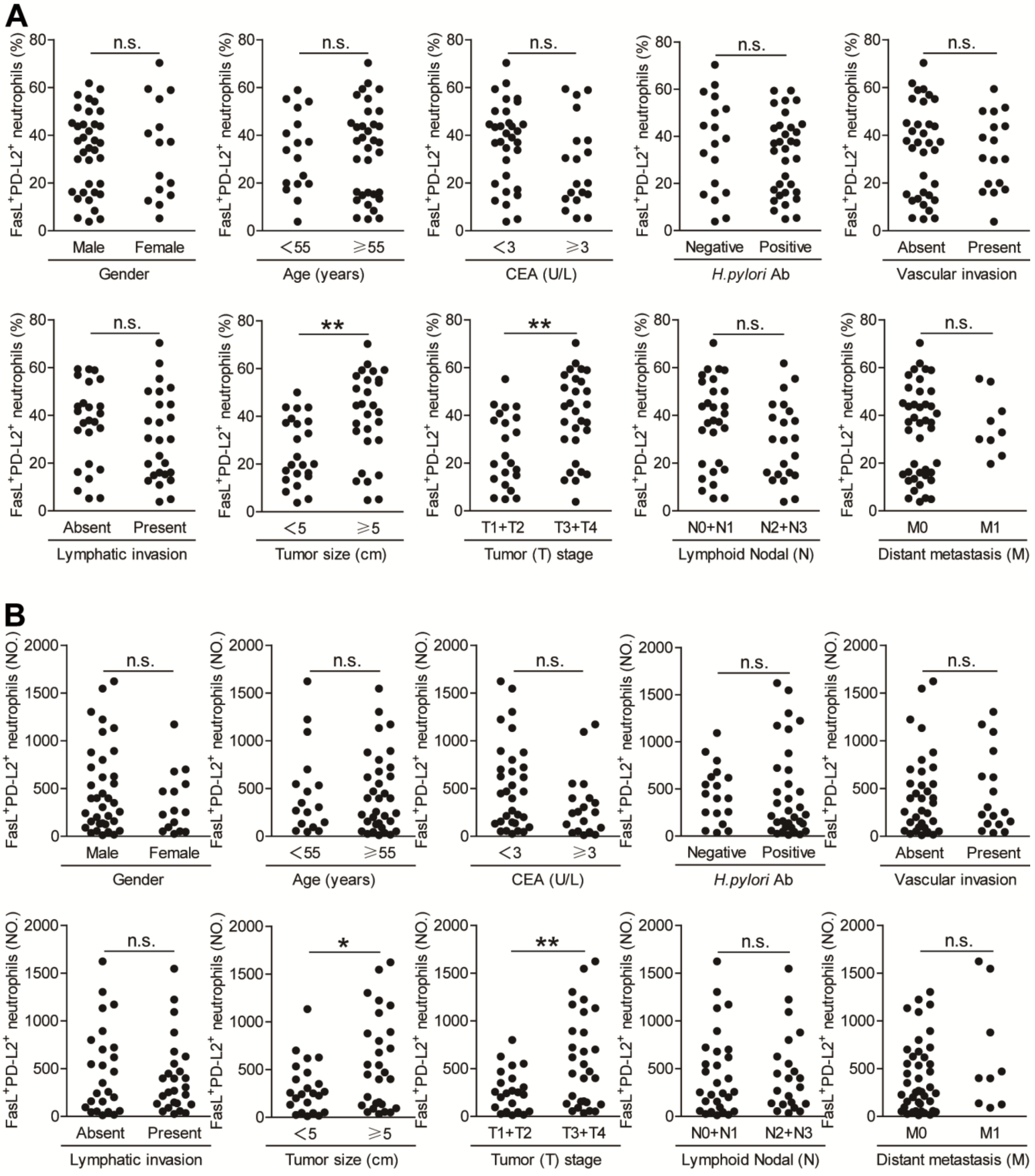

Figure S4. FasL$^+$PD-L2$^+$ neutrophils and their potential correlations with clinical parameters (cohort 1). (A and B) FasL$^+$PD-L2$^+$ neutrophil percentage in neutrophils (A) or FasL$^+$PD-L2$^+$ neutrophil number per million total cells (B) was analyzed for correlations with clinical pathological parameters. Data are analyzed by Student *t* test and Mann-Whitney U test. **P*<0.05, ***P*<0.01, n.s *P*>0.05 for groups connected by horizontal lines. CEA, carcinoembryonic antigen; *H.pylori* Ab, *Helicobacter pylori* antibody.

Figure S5.

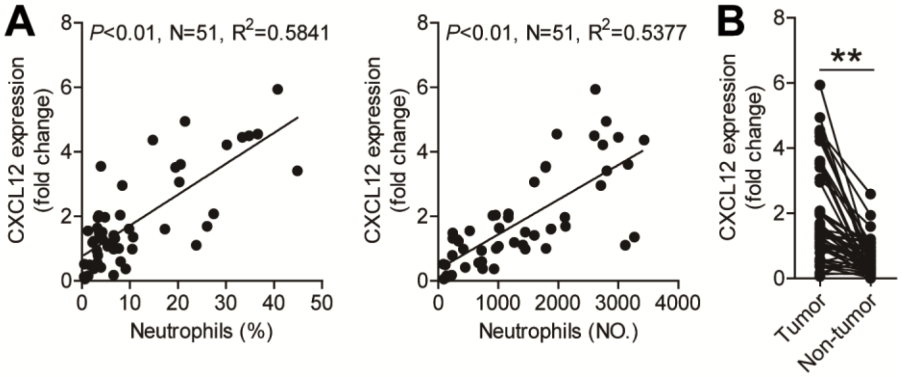

Figure S5. Increased neutrophil accumulation in GC tumors is promoted by CXCL12-CXCR4-mediated chemotaxis. (A) The correlations between neutrophils and CXCL12 in GC tumors were analyzed. Results were expressed as neutrophil percentage in $CD45^+$ leukocytes or neutrophil number per million total cells and CXCL12 expression in tumor tissues. (B) CXCL12 expression between autologous tumor and non-tumor tissues (n=51) was analyzed. Data are analyzed by Student *t* test and Mann-Whitney U test. **P*<0.05, ***P*<0.01, n.s *P*>0.05 for groups connected by horizontal lines.

Figure S6.

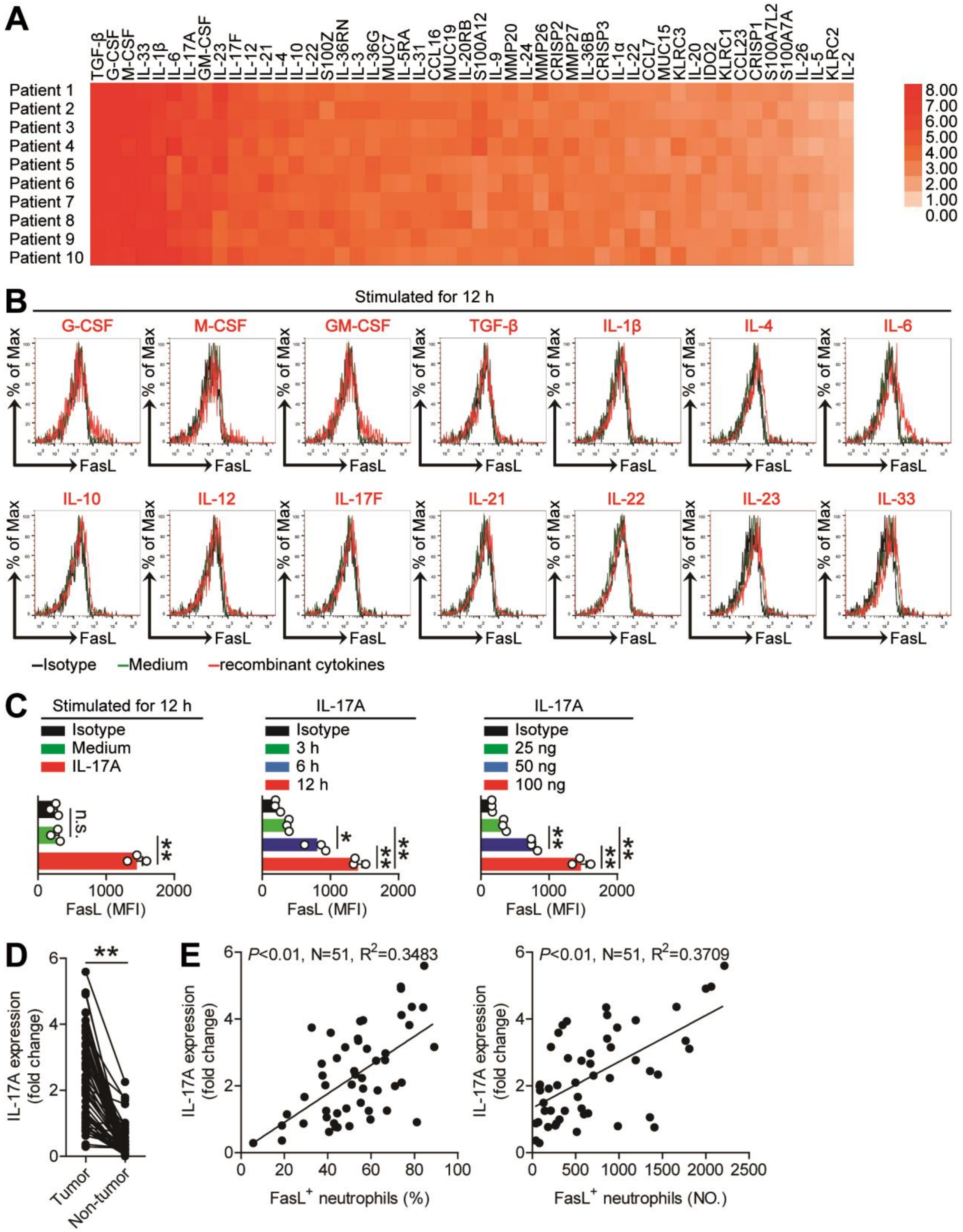

Figure S6. IL-17A induces neutrophil FasL expression. (A) Clustering of microarray data for the expression of 50 pro-inflammatory cytokine genes in human tumor tissues from 10 GC patients. (B) Expression of FasL on neutrophils exposed to G-CSF, M-CSF, GM-CSF, TGF-β, IL-1β, IL-4, IL-6, IL-10, IL-12, IL-17F, IL-21, IL-22, IL-23, IL-33 (100 ng/ml) for 12 h. (C) Statistical analysis of the expression of FasL on neutrophils exposed to IL-17A (100 ng/ml) or medium control for 12 h, or

exposed to IL-17A (100 ng/ml) for 3, 6, 12 h, or exposed to IL-17A (25, 50, or 100 ng/ml) for 12 h (n=3). (D) IL-17A expression between autologous tumor and non-tumor tissues (n=51) was analyzed. (E) The correlations between $FasL^+$ neutrophils and IL-17A in GC tumors were analyzed. Results were expressed as $FasL^+$ neutrophil percentage in total neutrophils or $FasL^+$ neutrophil number per million total cells and IL-17A expression in tumor tissues. Data are mean ± SEM and analyzed by Student *t* test, Mann-Whitney U test and 1-way ANOVA. *$P<0.05$, **$P<0.01$, n.s $P>0.05$ for groups connected by horizontal lines.

Figure S7.

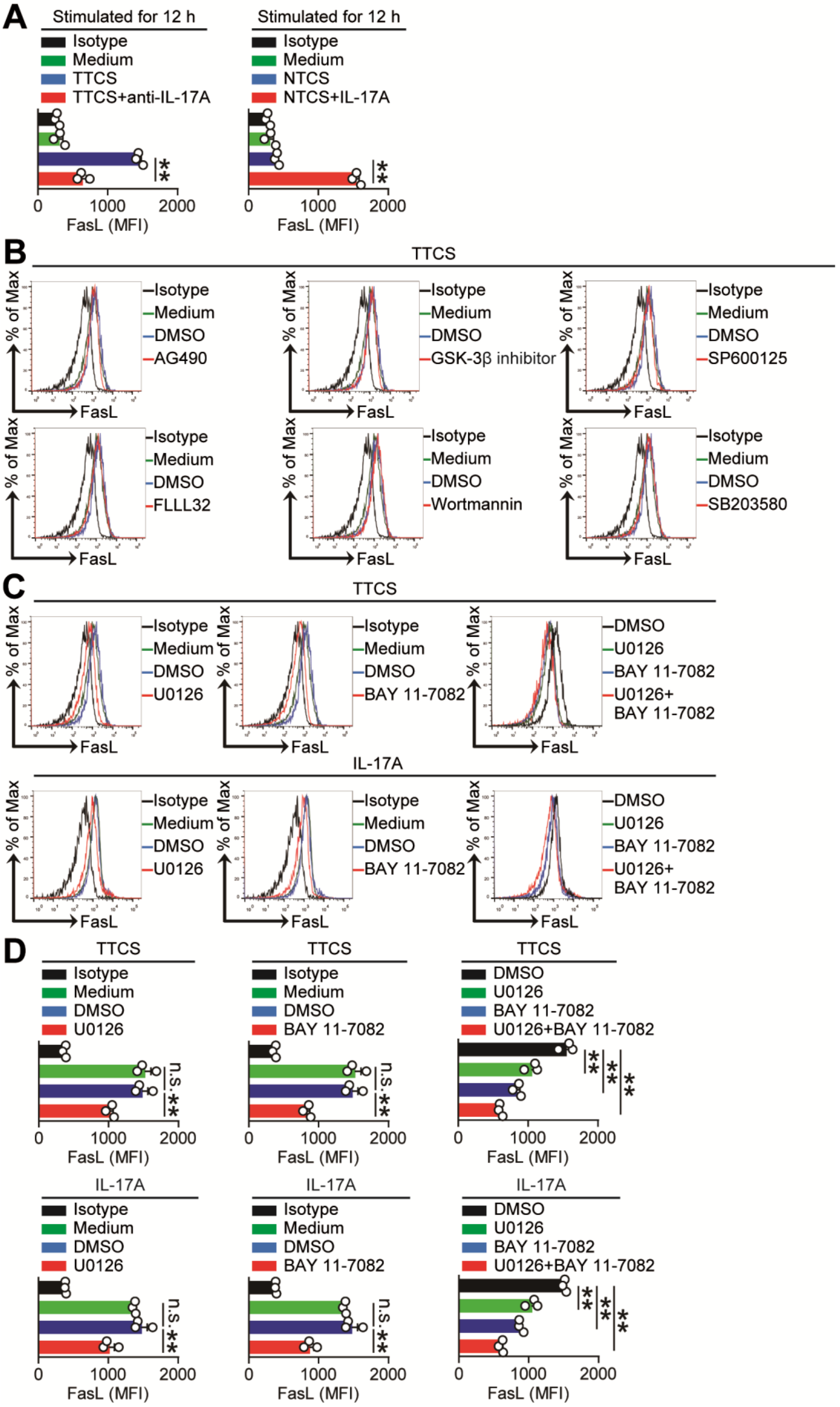

Figure S7. IL-17A induces neutrophil FasL expression via activating ERK-NF-κB signaling pathway. (A) Statistical analysis of the expression of FasL on neutrophils exposed to TTCS with

anti-IL-17A antibody or NTCS with IL-17A for 12 h (n=3). (B) Expression of FasL on neutrophils exposed to 50% TTCS with or without AG490 (a JAK inhibitor), SP600125 (a JNK inhibitor), FLLL32 (an STAT3 inhibitor), Wortmannin (a PI3K inhibitor), SB203580 (an MAPK inhibitor), or GSK-3β inhibitor for 12 h. (C) Expression of FasL on neutrophils exposed to TTCS or IL-17A with or without U0126 and/or BAY 11-7082 for 12 h. (D) Statistical analysis of the expression of FasL on neutrophils exposed to TTCS or IL-17A with or without U0126 and/or BAY 11-7082 for 12 h (n=3). Data are mean ± SEM and analyzed by Student *t* test, Mann-Whitney U test and 1-way ANOVA. *$P<0.05$, **$P<0.01$, n.s $P>0.05$ for groups connected by horizontal lines.

Figure S8.

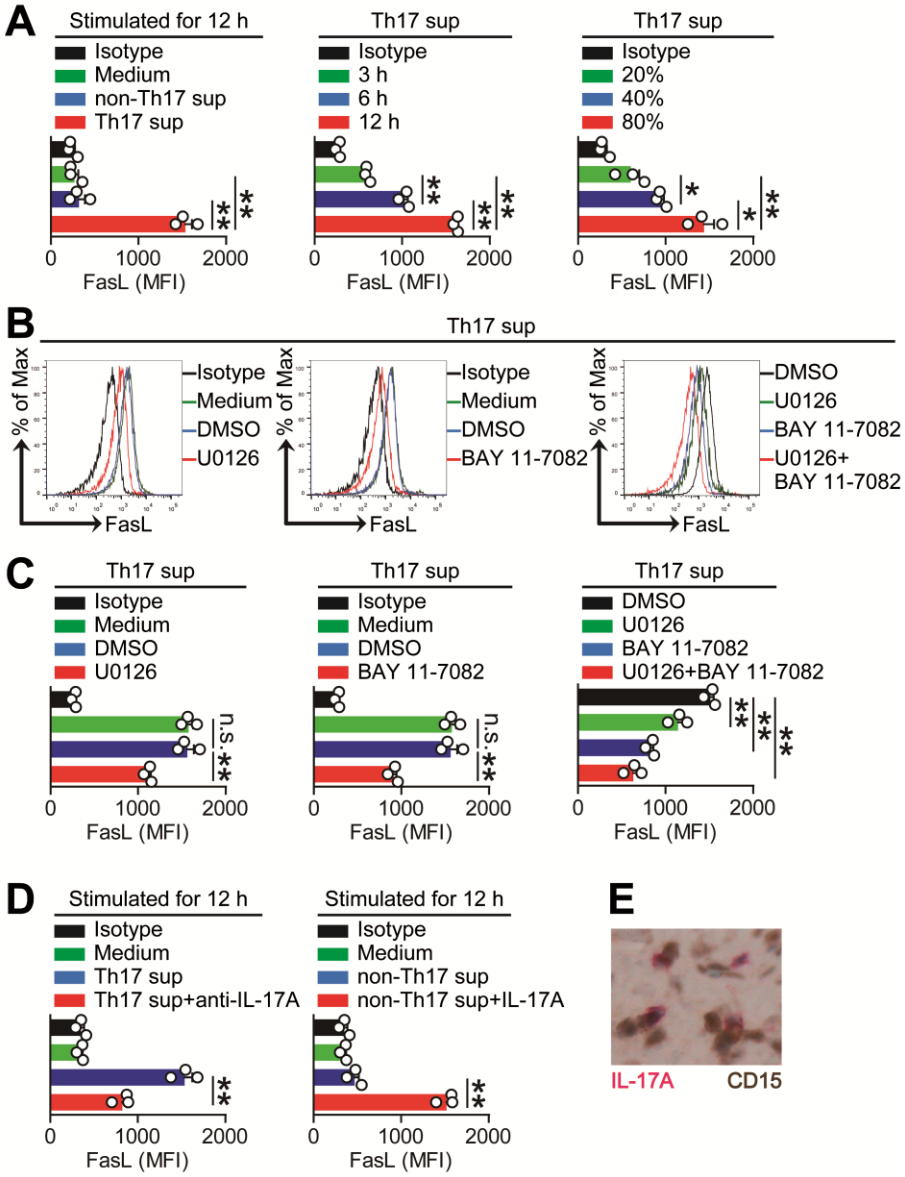

Figure S8. Th17 cell-derived IL-17A induces neutrophil FasL expression via activating ERK-NF-κB signaling pathway. (A) Statistical analysis of the expression of FasL on neutrophils exposed to Th17 sup, non-Th17 sup or medium control for 12 h, or exposed to Th17 sup for 3, 6, 12 h, or exposed to Th17 sup (20%, 40%, or 80%) for 12 h (n=3). (B) Expression of FasL on neutrophils exposed to Th17 sup with or without U0126 and/or BAY 11-7082 for 12 h. (C) Statistical analysis of the expression of FasL on neutrophils exposed to Th17 sup with or without U0126 and/or BAY 11-7082 for 12 h (n=3). (D) Statistical analysis of the expression of FasL on neutrophils exposed to Th17 sup with anti-IL-17A antibody or non-Th17 sup with IL-17A for 12 h (n=3). (E) Representative image of $CD15^+$ neutrophils and IL-$17A^+$ cells in tumor tissues of GC patients by immunohistochemical staining. Data are mean ± SEM and analyzed by Student *t* test, Mann-

Whitney U test and 1-way ANOVA. *P<0.05, **P<0.01, n.s P>0.05 for groups connected by horizontal lines. Th17 sup: Th17 cell culture supernatants; non-Th17 sup: non-Th17 cell culture supernatants.

Figure S9.

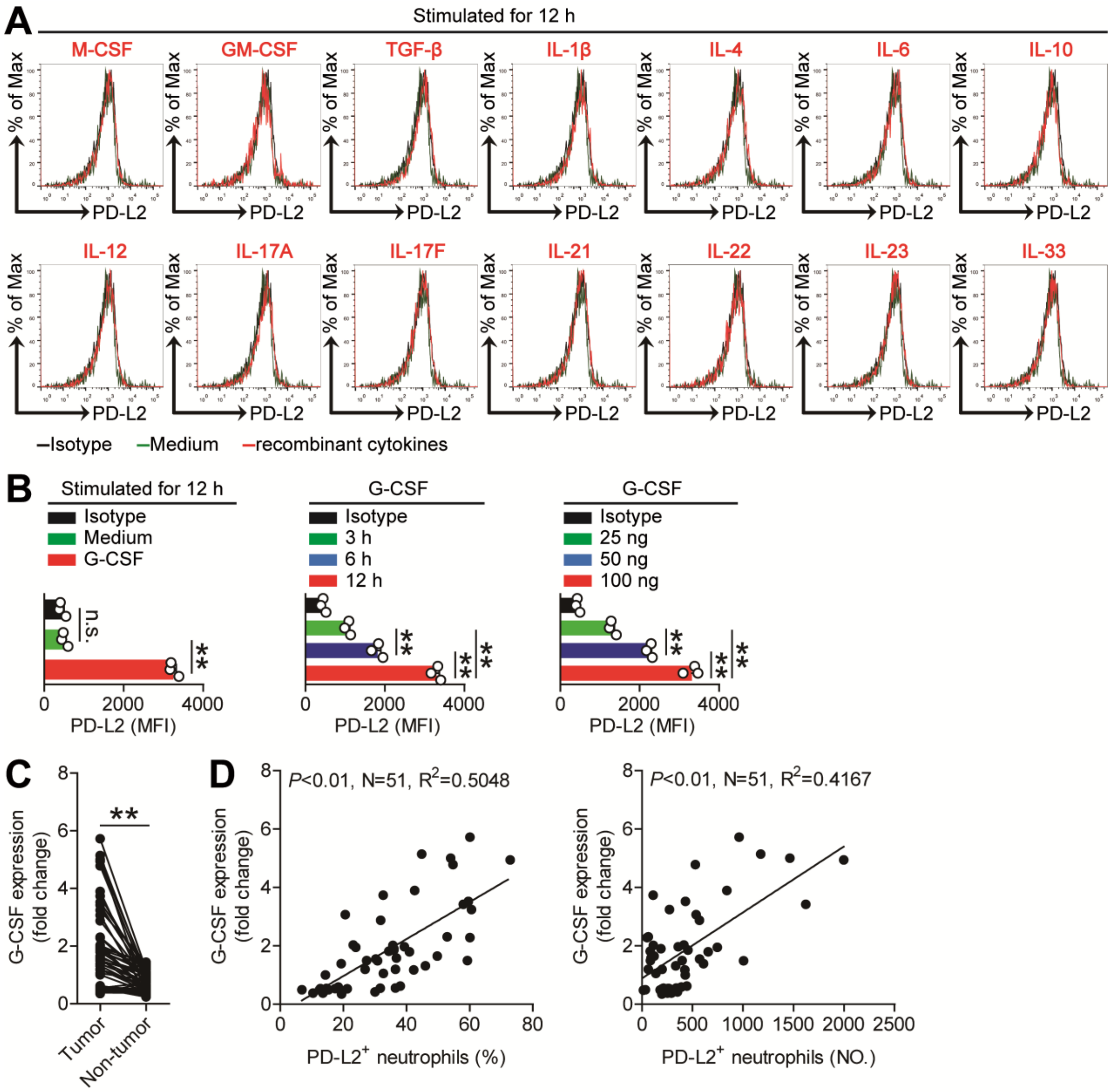

Figure S9. G-CSF induces neutrophil PD-L2 expression. (A) Expression of PD-L2 on neutrophils exposed to M-CSF, GM-CSF, TGF-β, IL-1β, IL-4, IL-6, IL-10, IL-12, IL-17A, IL-17F, IL-21, IL-22, IL-23, IL-33 (100 ng/ml) for 12 h. (B) Statistical analysis of the expression of PD-L2 on neutrophils exposed to G-CSF (100 ng/ml) or medium control for 12 h, or exposed to G-CSF (100 ng/ml) for 3, 6, 12 h, or exposed to G-CSF (25, 50, or 100 ng/ml) for 12 h (n=3). (C) G-CSF expression between autologous tumor and non-tumor tissues (n=51) was analyzed. (D) The correlations between PD-L2$^+$ neutrophils and G-CSF in GC tumors were analyzed. Results were expressed as PD-L2$^+$ neutrophil percentage in total neutrophils or PD-L2$^+$ neutrophil number per million total cells and G-CSF expression in tumor tissues. Data are mean ± SEM and analyzed by Student *t* test, Mann-Whitney U test and 1-way ANOVA. **P*<0.05, ***P*<0.01, n.s *P*>0.05 for groups connected by horizontal lines.

Figure S10.

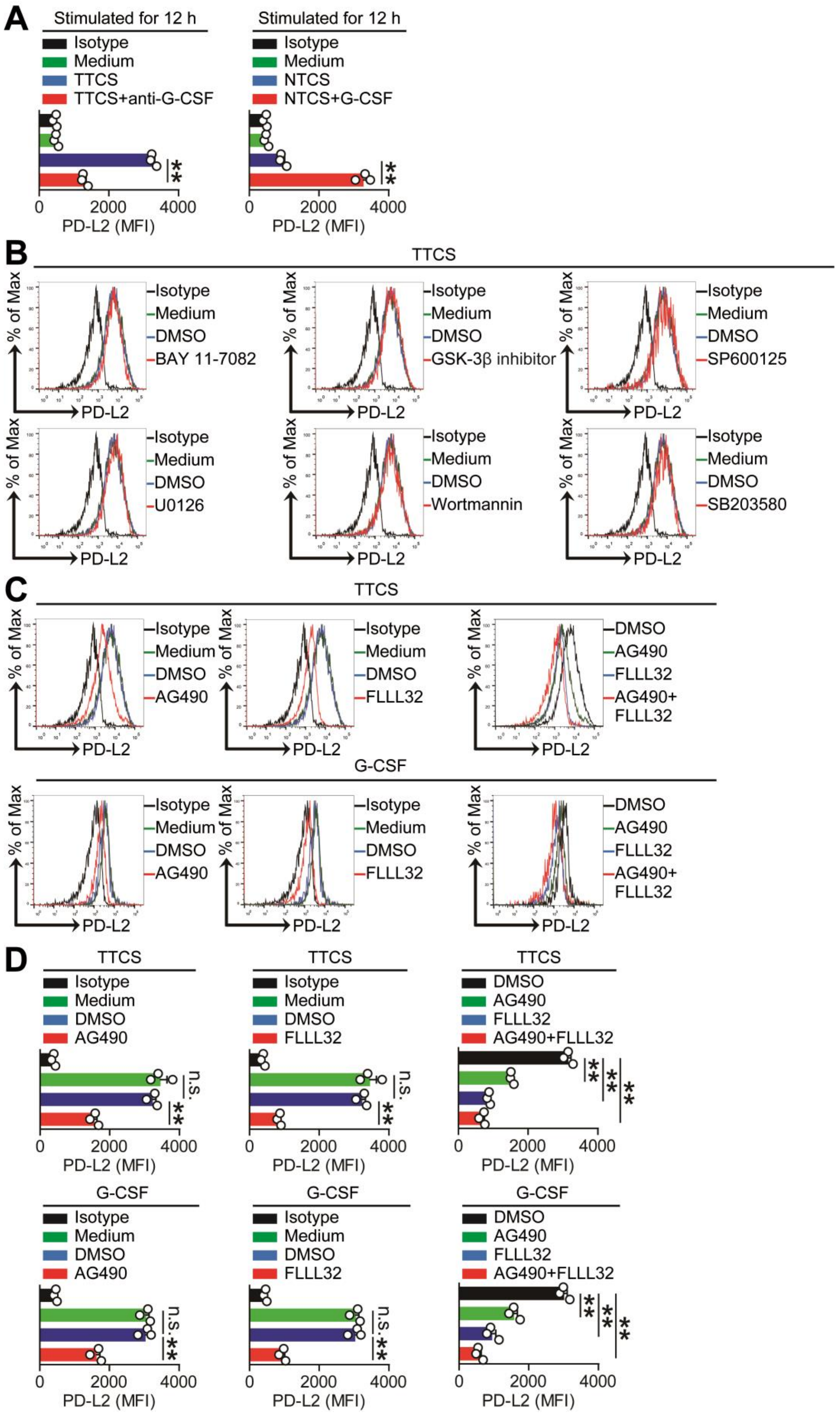

Figure S10. G-CSF induces neutrophil PD-L2 expression via activating JAK-STAT3 signaling pathway. (A) Statistical analysis of the expression of PD-L2 on neutrophils exposed to TTCS with

anti-G-CSF antibody or NTCS with G-CSF for 12 h (n=3). (B) Expression of PD-L2 on neutrophils exposed to 50% TTCS with or without BAY 11-7082 (an IκBα inhibitor), SP600125 (a JNK inhibitor), U0126 (an ERK inhibitor), Wortmannin (a PI3K inhibitor), SB203580 (an MAPK inhibitor), or GSK-3β inhibitor for 12 h. (C) Expression of PD-L2 on neutrophils exposed to TTCS or G-CSF with or without AG490 and/or FLLL32 for 12 h. (D) Statistical analysis of the expression of PD-L2 on neutrophils exposed to TTCS or G-CSF with or without AG490 and/or FLLL32 for 12 h (n=3). Data are mean ± SEM and analyzed by Student *t* test, Mann-Whitney U test and 1-way ANOVA. *$P<0.05$, **$P<0.01$, n.s $P>0.05$ for groups connected by horizontal lines.

Figure S11.

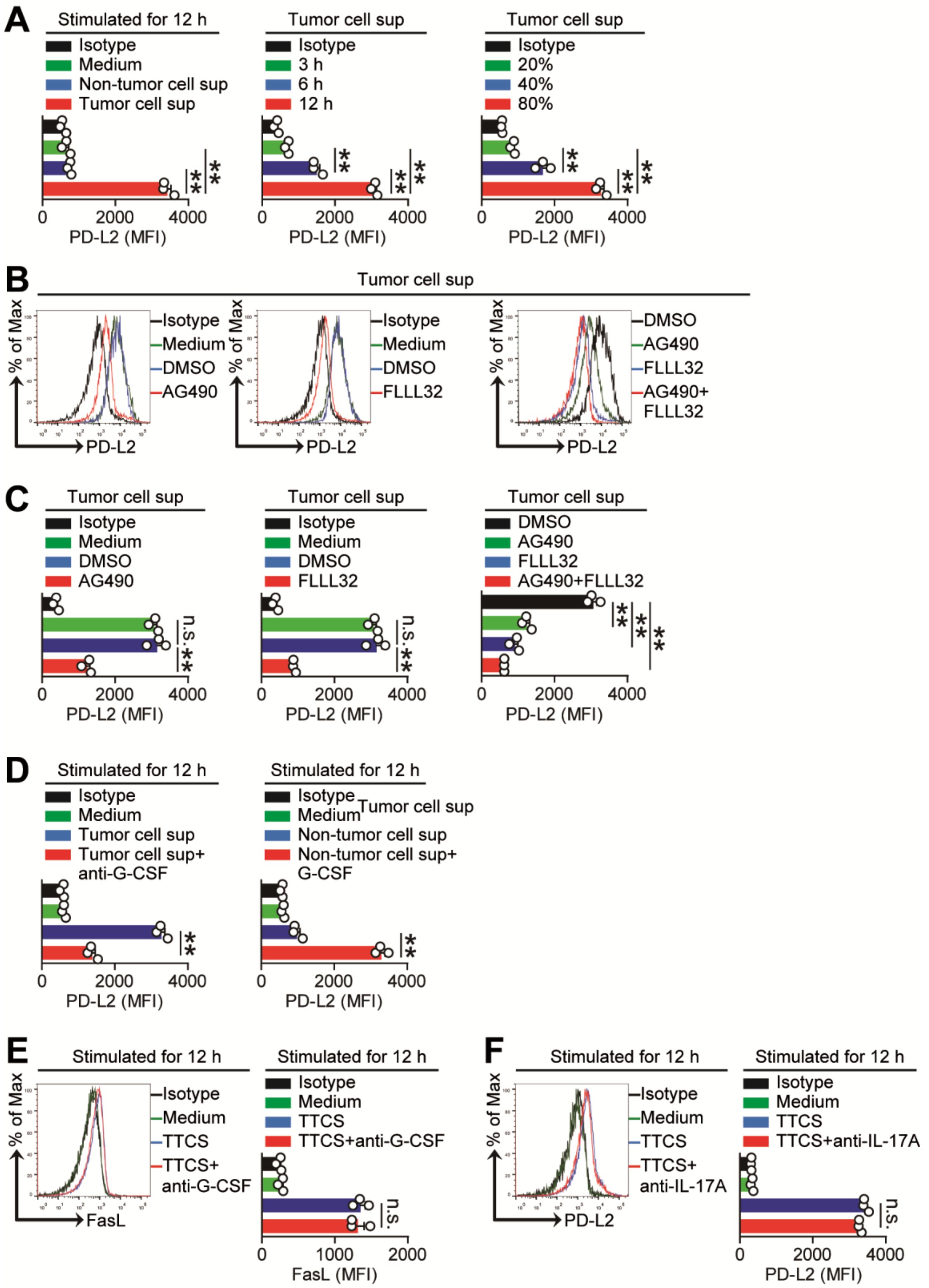

Figure S11. Tumor cell-derived G-CSF induces neutrophil PD-L2 expression via activating JAK-STAT3 signaling pathway. (A) Statistical analysis of the expression of PD-L2 on neutrophils exposed to tumor cell sup, non-tumor cell sup or medium control for 12 h, or exposed to tumor cell sup for 3, 6, 12 h, or exposed to tumor cell sup (20%, 40%, or 80%) for 12 h (n=3). (B) Expression of PD-L2 on neutrophils exposed to tumor cell sup with or without AG490 and/or FLLL32 for 12 h. (C) Statistical analysis of the expression of PD-L2 on neutrophils exposed to tumor cell sup with or

without AG490 and/or FLLL32 for 12 h (n=3). (D) Statistical analysis of the expression of PD-L2 on neutrophils exposed to tumor cell sup with anti-G-CSF antibody or non-tumor cell sup with G-CSF for 12 h (n=3). (E) Representative data and statistical analysis of the expression of FasL on neutrophils exposed to TTCS with anti-G-CSF antibody for 12 h (n=3). (F) Representative data and statistical analysis of the expression of PD-L2 on neutrophils exposed to TTCS with anti-IL-17A antibody for 12 h (n=3). Data are mean ± SEM and analyzed by Student *t* test, Mann-Whitney U test and 1-way ANOVA. *P<0.05, **P<0.01, n.s P>0.05 for groups connected by horizontal lines. Tumor cell sup: tumor cell culture supernatants; Non-tumor cell sup: non-tumor cell culture supernatants.

Figure S12.

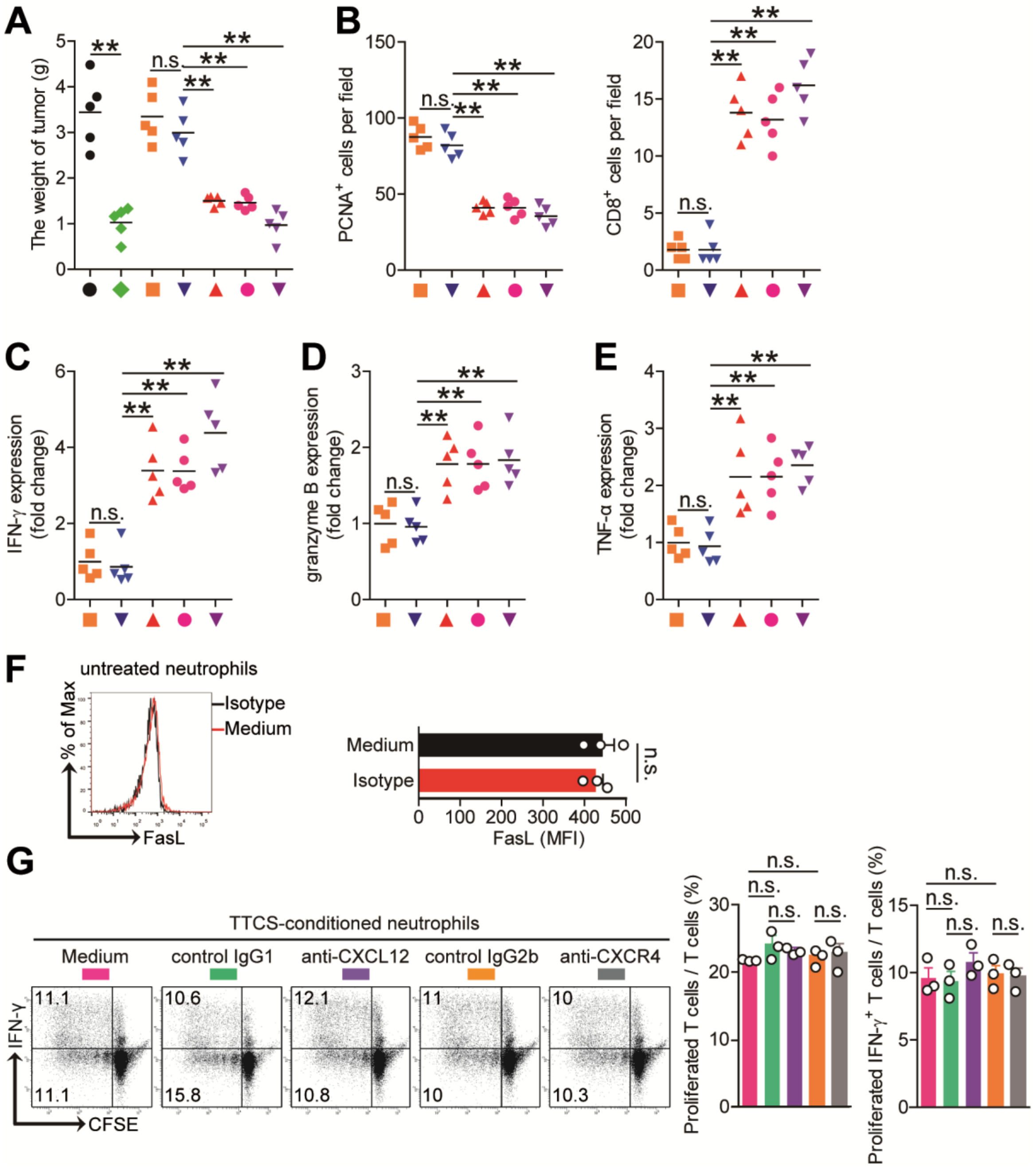

Figure S12. Blockade of neutrophil-associated FasL and PD-L2 on tumor-specific CD8$^+$ T-cell immunity inhibits tumor growth and GC progression. (A) Mice were injected with human SGC-7901 cells, as described in Methods. The control animals received no further injections. The experimental treatments entailed injections with tumor-specific CD8$^+$ T cells in combination with untreated neutrophils (N) or TTCS-conditioned neutrophils (TCN), or TCN pretreated with anti-FasL and/or anti-PD-L2 antibody or a control IgG. Statistical analysis of tumor weight on day 28 after tumor cell injection (n=5). (B) Statistical analysis of the expression of proliferating cell nuclear antigen (PCNA) or the infiltration of CD8$^+$ T cells in tumors of mice injected with tumor-specific CD8$^+$ T cells in combination with TTCS-conditioned neutrophils (TCN), or TCN pretreated with anti-FasL and/or anti-PD-L2 antibody or a control IgG on day 28 after tumor cell injection

(n=5). (C-E) IFN-γ expression (C), granzyme B expression (D) and TNF-α expression (E) in tumors of mice injected with tumor-specific $CD8^+$ T cells in combination with TTCS-conditioned neutrophils (TCN), or TCN pretreated with anti-FasL and/or anti-PD-L2 antibody or a control IgG on day 28 after tumor cell injection were compared (n=5). (F) Representative data and statistical analysis of the expression of FasL on untreated neutrophils (n=3). (G) CFSE-labeled tumor-specific $CD8^+$ T cells of donors were co-cultured for 5 days with autologous TTCS-conditioned neutrophils with or without anti-CXCR4 and/or anti-CXCL12 antibodies. Representative data and statistical analysis of T cell proliferation and proliferated IFN-γ-producing T cells were shown (n=3). Data are mean ± SEM and analyzed by Student *t* test, Mann-Whitney U test and 1-way ANOVA. *$P<0.05$, **$P<0.01$, n.s $P>0.05$ for groups connected by horizontal lines.

Figure S13.

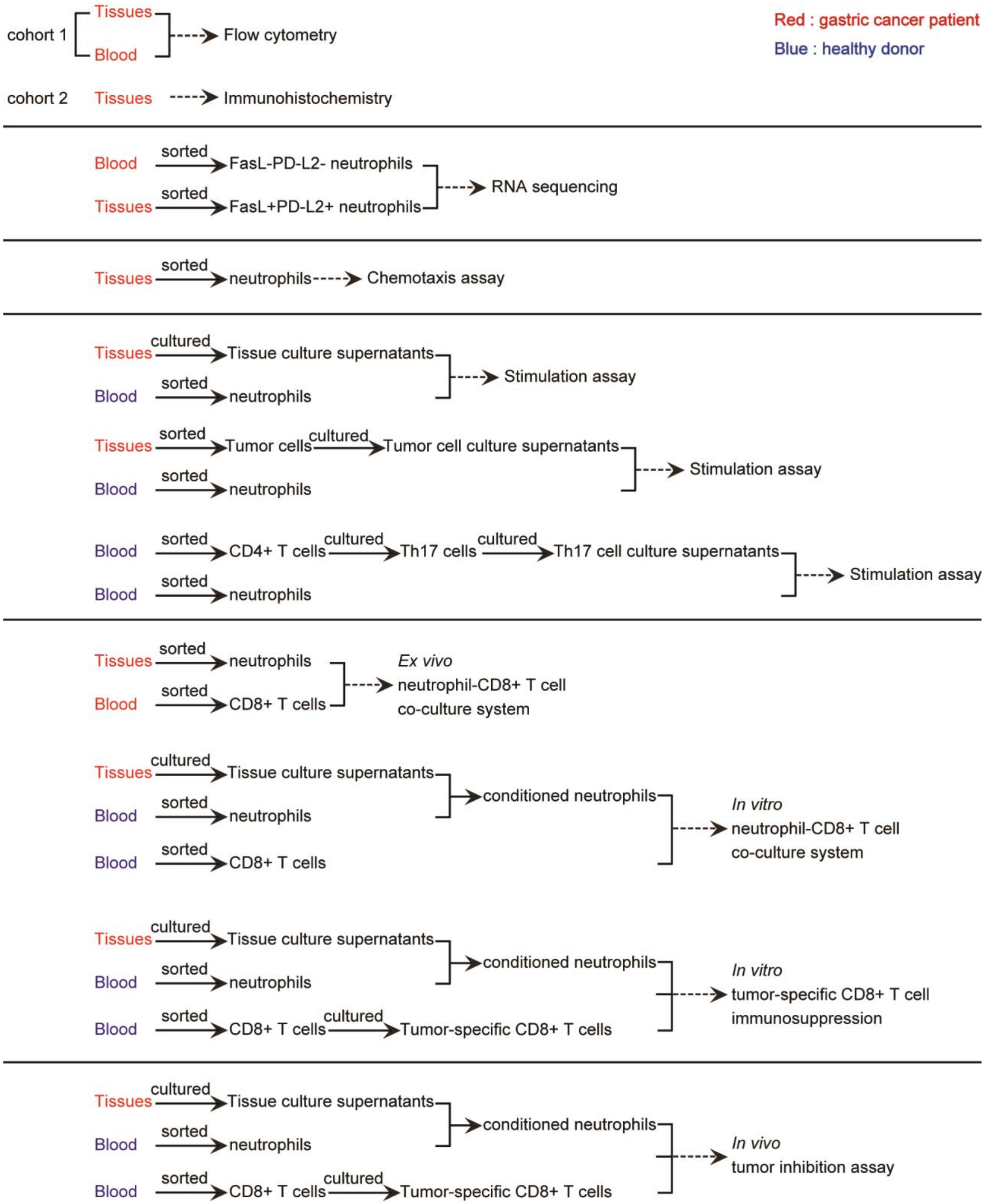

Figure S13. A schematic diagram outlining the experimental design is shown.

Table S1. Univariate and multivariate analyses of factors associated with overall survival

Variables	Univariate		Multivariate	
	P-value	HR	95% CI	P-value
Gender (male vs. female)	0.905			NA
Age, years (≥ 55 vs. < 55)	0.792			NA
H.pylori Ab (positive vs. negative)	0.512			NA
CEA,U/L (≥ 3 vs. < 3)	0.798			NA
Tumor size, cm (≥ 5 vs. < 5)	0.001			0.574
Lymphatic invasion (positive vs. negative)	0.014			0.199
Vascular invasion (positive vs. negtive)	0.052			NA
Tumor (T) invasion (T1+T2 vs. T3+T4)	<0.001			0.721
Lymphoid Nodal (N) status (N0+N1 vs. N2+N3)	0.004			0.392
Distant metastasis (M) status (M0 vs. M1)	0.175			NA
TNM stage (I+II vs. III+IV)	<0.001	2.359	1.158-4.805	0.018
Neutrophil numbera (high vs. low)	<0.001	1.005	1.002-1.008	0.003

Cox proportional hazards regression model. Variables used in multivariate analysis were adopted by univariate analysis. aNeutrophil number was acquired by immunohistochemical staining and counting and was expressed as number per field. CEA, carcinoembryonic antigen; H.pylori Ab, Helicobacter pylori antibody; HR, hazard ratio; CI, confidence interval; NA, not adopted.

Table S2. Univariate and multivariate analyses of factors associated with disease-free survival

Variables	Univariate	Multivariate		
	P-value	HR	95% CI	P-value
Gender (male vs. female)	0.956			NA
Age, years (≥ 55 vs. < 55)	0.937			NA
H.pylori Ab (positive vs. negative)	0.608			NA
CEA,U/L (≥ 3 vs. < 3)	0.496			NA
Tumor size, cm (≥ 5 vs. < 5)	0.001			NA
Lymphatic invasion (positive vs. negative)	0.007			NA
Vascular invasion (positive vs. negtive)	0.019			NA
Tumor (T) invasion (T1+T2 vs. T3+T4)	<0.001			NA
Lymphoid Nodal (N) status (N0+N1 vs. N2+N3)	0.003			NA
Distant metastasis (M) status (M0 vs. M1)	0.236			NA
TNM stage (I+II vs. III+IV)	<0.001	2.661	1.343-5.273	0.005
Neutrophil numbera (high vs. low)	<0.001	1.005	1.002-1.008	0.001

Cox proportional hazards regression model. Variables used in multivariate analysis were adopted by univariate analysis. aNeutrophil number was acquired by immunohistochemical staining and counting and was expressed as number per field. CEA, carcinoembryonic antigen; H.pylori Ab, Helicobacter pylori antibody; HR, hazard ratio; CI, confidence interval; NA, not adopted.

Table S3. Univariate and multivariate analyses of factors associated with survival

Variables	Univariate	Multivariate		
	P-value	HR	95% CI	P-value
Gender (male vs. female)	0.875			NA
Age, years (≥ 55 vs. < 55)	0.191			NA
H.pylori Ab (positive vs. negative)	0.784			NA
CEA,U/L (≥ 3 vs. < 3)	0.224			NA
Tumor size, cm (≥ 5 vs. < 5)	0.055			NA
Lymphatic invasion (positive vs. negative)	0.447			NA
Vascular invasion (positive vs. negtive)	0.857			NA
Tumor (T) invasion (T1+T2 vs. T3+T4)	0.075			NA
Lymphoid Nodal (N) status (N0+N1 vs. N2+N3)	0.110			NA
Distant metastasis (M) status (M0 vs. M1)	0.779			NA
TNM stage (I+II vs. III+IV)	0.011	4.393	0.938-20.572	0.060
FasL+PD-L2+ neutrophil percentagea (high vs. low)	0.016	1.150	0.375-3.520	0.807
FasL+PD-L2+ neutrophil numberb (high vs. low)	<0.001	5.461	1.486-20.066	0.011

Cox proportional hazards regression model. Variables used in multivariate analysis were adopted by univariate analysis. aFasL+PD-L2+ neutrophil percentage was acquired on CD45+CD11b+CD66b+CD15+FasL+PD-L2+ cells that gated on CD45+CD11b+CD66b+CD15+ cells of tumor tissues. bFasL+PD-L2+ neutrophil number was acquired by counting CD45+CD11b+CD66b+CD15+FasL+PD-L2+ cells per million cells of tumor tissues. CEA, carcinoembryonic antigen; H.pylori Ab, Helicobacter pylori antibody; HR, hazard ratio; CI, confidence interval; NA, not adopted.

Table S4. Clinical characteristics of 51 patients with gastric cancer (cohort 1)

Variables	No. of patients
Gender (male/female)	36/15
Age (years; median, range)	61, 24-78
H.pylori Ab (negative/positive)	18/33
CEA (U/L; <3/≥3)	32/19
Tumor size (cm; <5/≥5)	23/28
Lymphatic invasion (absent/present)	25/26
Vascular invasion (absent/present)	34/17
Tumor (T) invasion (T1+T2/T3+T4)	22/29
Lymphoid Nodal (N) status (N0+N1/N2+N3)	29/22
Distant metastasis (M) status (M0/M1)	42/9
TNM stage (I+II/III+IV)	19/32
Neutrophil percentagea (median, range)	6.66, 0.46-44.87
Neutrophil numberb (median, range)	1171, 85-3431
FasL+PD-L2+ neutrophil percentagec (median, range)	36.8, 3.85-70.3
FasL+PD-L2+ neutrophil numberd (median, range)	306, 13-1623

aNeutrophil percentage was acquired on CD45+CD11b+CD66b+CD15+ cells that gated on CD45+ cells of tumor tissues. bNeutrophil number was acquired by counting CD45+CD11b+CD66b+CD15+ cells per million cells of tumor tissues. cFasL+PD-L2+ neutrophil percentage was acquired on CD45+CD11b+CD66b+CD15+FasL+PD-L2+ cells that gated on CD45+CD11b+CD66b+CD15+ cells of tumor tissues. dFasL+PD-L2+ neutrophil number was acquired by counting CD45+CD11b+CD66b+CD15+FasL+PD-L2+ cells per million cells of tumor tissues. CEA, carcinoembryonic antigen; H.pylori Ab, Helicobacter pylori antibody.

Table S5. Clinical characteristics of 125 patients with gastric cancer (cohort 2)

Variables	No. of patients
Gender (male/female)	93/32
Age (years; median, range)	56, 25-82
H.pylori Ab (negative/positive)	23/102
CEA (U/L; <3/≥3)	99/26
Tumor size (cm; <5/≥5)	61/64
Lymphatic invasion (absent/present)	45/80
Vascular invasion (absent/present)	114/11
Tumor (T) invasion (T1+T2/T3+T4)	42/83
Lymphoid Nodal (N) status (N0+N1/N2+N3)	56/69
Distant metastasis (M) status (M0/M1)	109/16
TNM stage (I+II/III+IV)	40/85
Neutrophil numbera (median, range)	132, 28-385

aNeutrophil number was acquired by immunohistochemical staining and counting and was expressed as number per field. CEA, carcinoembryonic antigen; H.pylori Ab, Helicobacter pylori antibody.

Table S6. Antibodies and other reagents

Antibodies and reagents	Manufacturers
Antibodies for flow cytometry	
anti-CD45-PE-Cy7	Biolegend
anti-CD11b-PerCP-Cy5.5	Biolegend
anti-CD66b-FITC	Biolegend
anti-CD15-APC-Cy7	Biolegend
anti-FasL-PE	Biolegend
anti-PD-L2-Alexa Fluor® 647	Biolegend
anti-CXCR4-APC	Biolegend
anti-CD3-APC-Cy7	BD Pharmingen
anti-CD8-PerCP-Cy5.5	Biolegend
anti-IFN-γ-PE-Cy7	BD Pharmingen
anti-granzyme B-Alexa Fluor® 647	Biolegend
anti-TNF-α-FITC	Biolegend
Antibodies for immunohistochemical staining	
anti-human CD15	Abcam
anti-human MPO	Abcam
anti-human FasL	Abcam
anti-human PD-L2	Abcam
anti-human IL-17A	Abcam
anti-human CD8	Abcam
anti-human proliferating cell nuclear antigen (PCNA)	Abcam
horseradish peroxidase anti-rabbit IgG	Zhongshan Biotechnology
horseradish peroxidase anti-mouse IgG	Zhongshan Biotechnology
DAB kit	Zhongshan Biotechnology
EnVision™ G2 System/AP Rabbit/Mouse (Permanent Red)	Dako
Antibodies for immunofluorescence	
rabbit anti-human CD15	Abcam
mouse anti-human CD8	Abcam
rabbit anti-human G-CSF	Abcam
mouse anti-human EpCam	Abcam
goat anti-rabbit-FITC	Zhongshan Biotechnology
goat anti-mouse-TRITC	Zhongshan Biotechnology
Antibodies for neutralizing and blocking	
anti-human CXCL12 (Mouse IgG1)	R&D Systems
Mouse IgG1 Isotype Control	R&D Systems
anti-human CXCR4 (Mouse IgG2b)	R&D Systems

Mouse IgG2b Isotype Control	R&D Systems
anti-human G-CSF (Mouse IgG1)	R&D Systems
Mouse IgG1 Isotype Control	R&D Systems
anti-human IL-17A (Rabbit IgG)	Abcam
Rabbit IgG Control	Abcam
anti-human FasL (Mouse IgG2b)	R&D Systems
Mouse IgG2b Isotype Control	R&D Systems
anti-human PD-L2 (Goat IgG)	R&D Systems
Goat IgG Control	R&D Systems
Antibodies for western blot	
anti-human p-p65	Abcam
anti-human p65	Abcam
anti-STAT3	Abcam
anti-p-STAT3	Abcam
anti-human GAPDH	Abcam
Purified anti-CD3 and anti-CD28 antibodies	Biolegend
ELISA kits	
G-CSF	R&D Systems
TNF-α	R&D Systems
granzyme B	eBioscience
IFN-γ	Abcam
CXCL12	Abcam
IL-17A	Abcam
Reagents for signaling pathways inhibition	
MEK-1 and MEK-2 inhibitor U0126	Merk Millipore
IκBα inhibitor BAY 11-7082	Calbiochem
JNK inhibitor SP600125	Calbiochem
MAPK inhibitor SB203580	Calbiochem
PI3K inhibitor Wortmannin	Calbiochem
STAT3 phosphorylation inhibitor FLLL32	MedKoo Biosciences
JAK signaling inhibitor AG490	Merk Millipore
GSK-3β inhibitor VI	Calbiochem
CD3 microbeads	Milteniy Biotec
EpCam microbeads	Milteniy Biotec
CD4 microbeads	StemCell Technologies
CD8 microbeads	StemCell Technologies
3-µm pore size Transwells	Corning
0.4-µm pore size Transwells	Corning

Collagenase IV	Gibco
DNase I	Sigma-Aldrich
Phorbol myristate acetate	Sigma-Aldrich
Ionomycin	Sigma-Aldrich
DMSO	Sigma-Aldrich
Golgistop and Perm/Wash solution	BD Pharmingen
Carboxylfluorescein succinimidyl ester (CFSE)	eBioscience
Protein Extraction Reagent	Pierce
SuperSignal® West Dura Extended Duration Substrate kit	Thermo
Fetal calf serum (FCS)	Gibco
Penicillin/Streptomycin	Gibco
RPMI-1640	Hyclone
Ficoll-Paque Plus	GE Healthcare
lyses solution	TIANGEN
TRIzol reagent	Invitrogen
PrimeScriptTM RT reagent Kit	TaKaRa
Real-time PCR Master Mix	Toyobo
human IL-17 Secretion Assay-Detection Kits	Miltenyi Biotec
All recombinant cytokines and chemokines	PeproTech

APC-Cy7, allophycocyanin-cyanin 7; PE-Cy7, phycoerythrin-cyanin 7; FITC, Fluorescein isothiocyanate; PE, phycoerythrin; PerCP-Cy5.5, peridin chlorophyl protein-cyanin 5.5; APC, allophycocyanin; IL, interleukin; PCNA, proliferating cell nuclear antigen; MPO, myeloperoxidase.

Table S7. Primer and probe sequences for real-time PCR analysis

Gene	Primer	Sequence 5′→3′
Human CXCL12	forward	TGGCGTGGAGCTGAGAGATAACC
	reverse	CGATGCGGCTGATGGTGTGG
Human IL-17A	forward	TCCAAGGTGGAAGTGGTAGC
	reverse	AGAAAACTGCTCCGCTGAAG
Human G-CSF	forward	CTGAGAGTGATTGAGAGTGG
	reverse	ACAACCCTCTGCACCCAGTT
Human IFN-γ	forward	GAGATATCCCTCTGTGATCTGG
	reverse	GACAGAGTTCATGTGGTAGTCC
Human granzyme B	forward	GAAAGTGCGAATCTGACTTACG
	reverse	TTGTTTCGTCCATAGGAGACAA
Human TNF-α	forward	TGGCGTGGAGCTGAGAGATAACC
	reverse	CGATGCGGCTGATGGTGTGG
Human GAPDH	forward	ACCCAGAAGACTGTGGATGG
	reverse	CAGTGAGCTTCCCGTTCAG
